# Revisiting the lipid ratio in lipid nanoparticles: A comparison of OFAT and design of experiments approaches

**DOI:** 10.1007/s13346-026-02125-6

**Published:** 2026-05-18

**Authors:** Jade Forrester, Eleanor Vaughan, Benjamin R. Knappett, Yvonne Perrie

**Affiliations:** 1 Strathclyde Institute of Pharmacy and Biomedical Sciences, University of Strathclyde, 161 Cathedral St, Glasgow, G4 0RE UK; 2 Unchained Labs Royston Ltd, Unit 1 Anglian Business Park, Royston, SG8 5TW UK

**Keywords:** Lipid nanoparticles, mRNA delivery, Lipid molar ratio, Formulation screening, One-factor-at-a-time, Design of Experiments, Microfluidic manufacturing, In vitro expression, In vivo protein expression

## Abstract

Despite the clinical success of lipid nanoparticles (LNPs), formulation optimisation remains dominated by inherited lipid ratios and screening approaches that fail to capture the combined influence of composition and manufacturing process. In this study, one-factor-at-a-time (OFAT) and Design of Experiments (DoE) strategies were applied to investigate how lipid molar ratio influences critical quality attributes (CQAs) and mRNA expression. In addition, manually executed and automated microfluidic manufacturing methods were compared to assess their impact on formulation–performance relationships. Across all strategies, multiple formulations beyond the standard molar ratio achieved comparable physicochemical properties, including particle size < 120 nm, low polydispersity index (PDI < 0.2), and high encapsulation efficiency (> 75%). Differences in CQAs and expression profiles were observed between manually and automated microfluidic manufactured DoE formulations, indicating that both composition and process conditions influence formulation performance. DoE analysis revealed non-linear and process-dependent relationships between lipid composition and CQAs, with limited predictive capability within the explored design space, particularly for biological outputs. Comparison of OFAT and DoE datasets demonstrated that while OFAT identifies directional trends within a constrained design space, DoE provides broader insight into interaction effects and formulation trade-offs. Overall, these findings demonstrate that the conventional lipid ratio represents a robust but not necessarily optimal formulation and support a shift towards context-dependent, design-space-driven optimisation of LNP systems.

**Graphical abstract:**

Schematic illustrating the experimental workflow used to compare one‑factor‑at‑a‑time (OFAT) and Design of Experiments (DoE) approaches for lipid nanoparticle (LNP) formulation development, including manual and automated microfluidic manufacture and subsequent physicochemical and biological evaluation.

**Figure:**
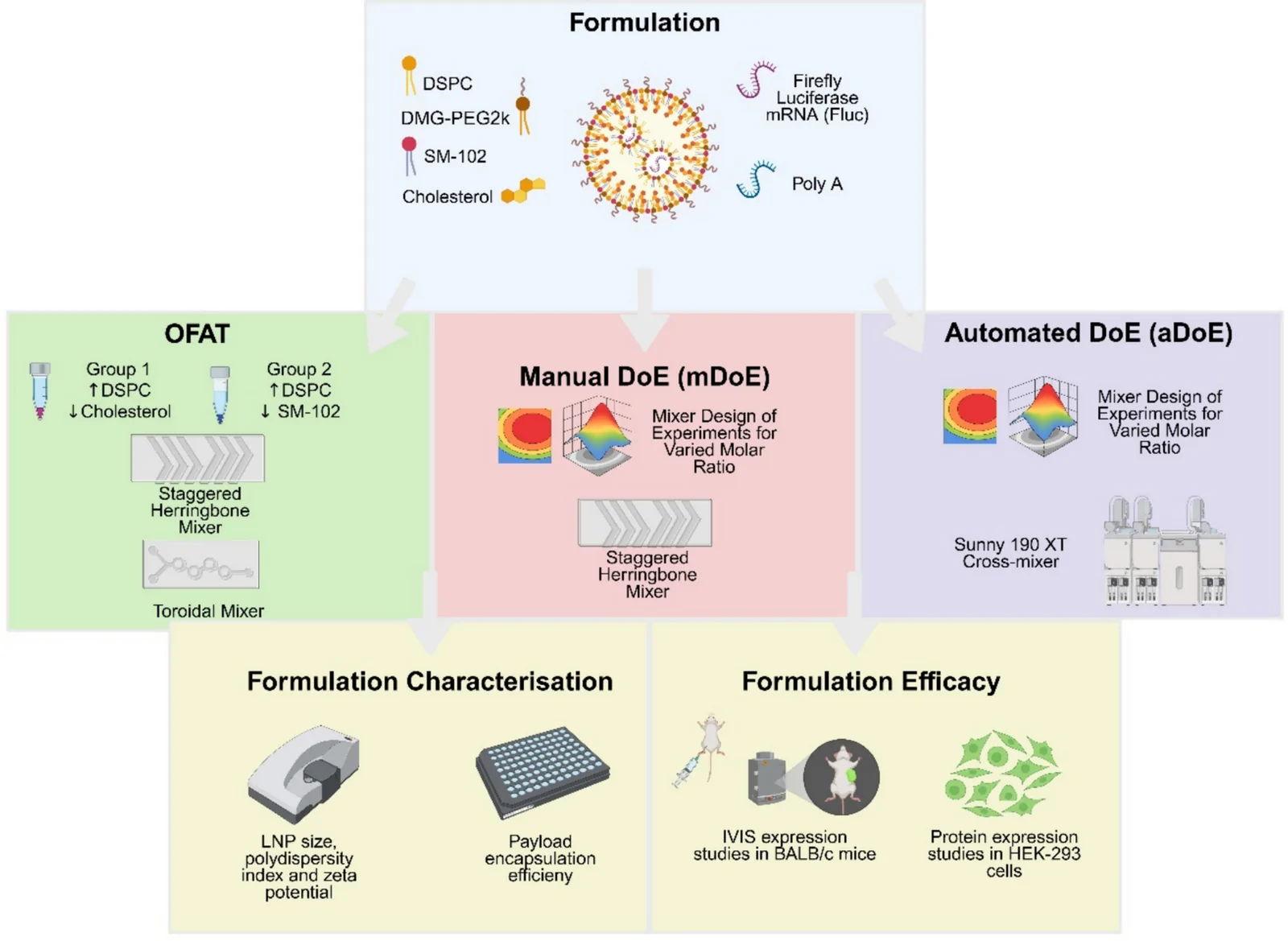

**Supplementary Information:**

The online version contains supplementary material available at https://doi.org/10.1007/s13346-026-02125-6.

## Introduction

Lipid nanoparticles (LNPs) are the most clinically advanced lipid-based nucleic acid therapeutics (NATs). They were developed to overcome key limitations of naked NATs, including rapid degradation and inefficient delivery of messenger RNA (mRNA) to target tissues [1]. Their clinical translation has been accelerated by incremental formulation development, culminating in the rapid deployment of LNP-based vaccines during the COVID-19 pandemic, supported by prior advances such as the development of Onpattro (Alnylam), the first siRNA-LNP formulation approved in 2018 [2].

The development of Onpattro provided a basis for many current LNP formulations, including Spikevax and mRESVIA. As a result, many contemporary mRNA-LNP systems are derived from a relatively narrow design space, with lipid compositions and molar ratios largely inherited from early siRNA formulations rather than systematically re-optimised for mRNA delivery. In general, for these LNPs, the current critical quality attribute (CQA) acceptance levels are typically defined as particle sizes <100 nm, polydispersity index <0.2, a near-neutral zeta potential, and high mRNA incorporation. These CQA thresholds, particularly LNP size, were primarily established to enable passage through liver fenestrae to facilitate hepatocyte targeting [3]. While appropriate for hepatic delivery, this design rationale may constrain formulation flexibility when alternative biodistribution or expression profiles are desired.

**Fig. 1:**
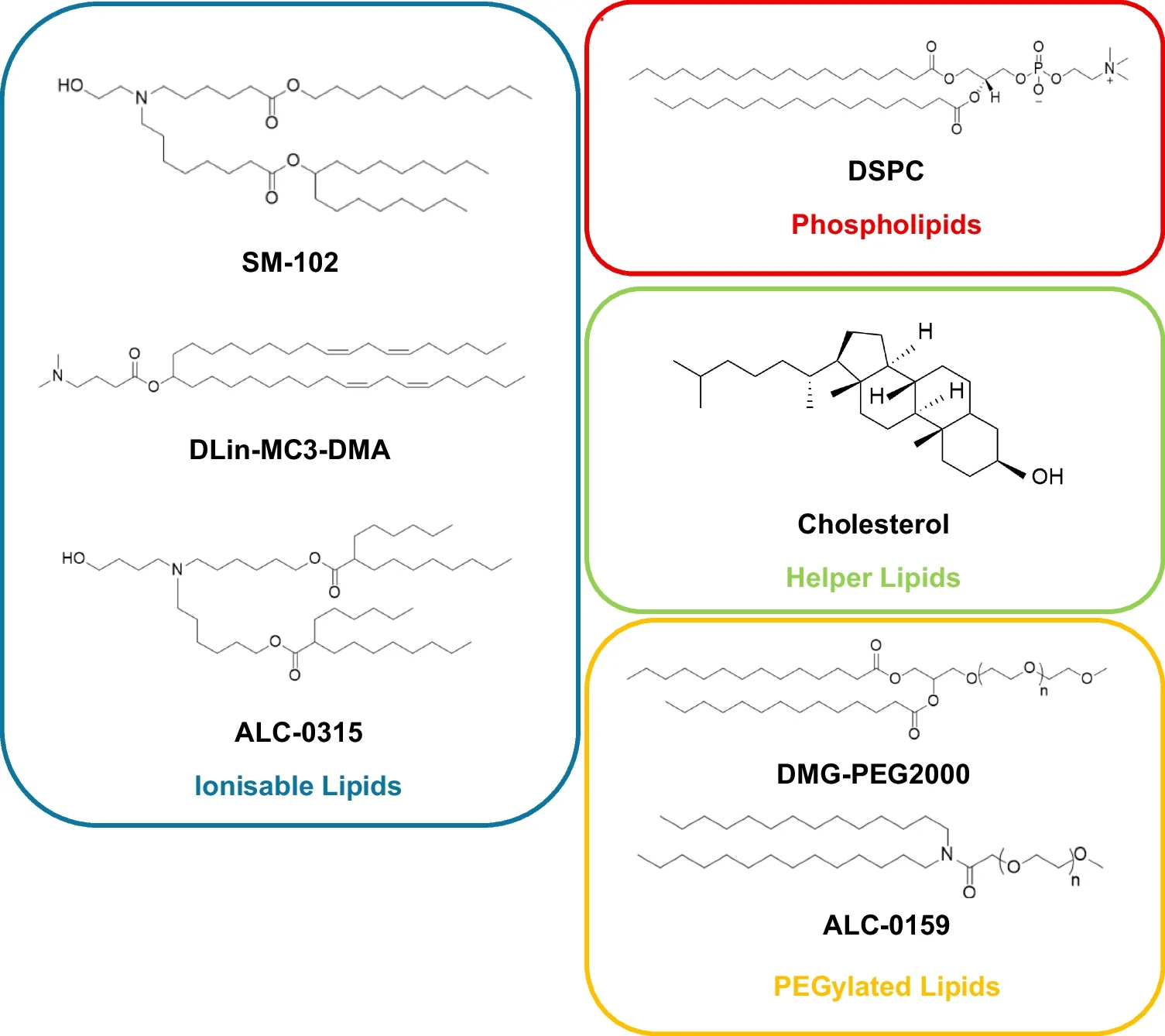
Chemical structures of lipids commonly used in clinically approved lipid nanoparticle (LNP) formulations. Ionisable lipids (SM-102, DLin-MC3-DMA and ALC-0315) are shown on the left, while helper lipids are shown on the right, including the phospholipid DSPC, cholesterol, and PEGylated lipids (DMG-PEG2000 and ALC-0159). Together, these four lipid classes form the basis of standard LNP formulations used for nucleic acid delivery. Structures were drawn using ChemDraw v23.1.2

Two key drivers that affect the CQAs of LNPs are the manufacturing method and the formulation composition, including the lipid components and their relative ratios. Typically, LNPs consist of a 4-lipid mixture at a standard molar ratio of 10% phospholipid, 38.5% cholesterol, 50% ionisable lipid and 1.5% PEGylated lipid [4]. Figure 1 illustrates the lipids included in the approved formulations, with Onpattro, Spikevax, and mRESVIA closely reflecting this composition, and Comirnaty having a similar ratio but with decreased phospholipid and ionisable lipid content in exchange for increased cholesterol (9.4:42.7:46.3:1.5%, respectively) [5]. Despite these relatively small differences, the overall formulation paradigm remains largely conserved across approved products. However, this consistency also highlights the limited systematic exploration of alternative molar ratios in current LNP design.

While changes in lipid composition may appear minimal, they can drive key characteristics and functional effects that can influence LNP efficiency. Importantly, the structural and functional behaviour of LNPs is not solely dictated by a single lipid component in isolation, but rather by complex, non-linear interactions between lipids. Therefore, it is vital to understand the role of each lipid and their combinatorial effects to better define the flexibility of the molar ratio and to enhance the effectiveness of mRNA-LNPs.

Phospholipids, commonly DSPC, are included within LNPs to increase stability and payload encapsulation by enhancing the interaction of the phosphate backbone of the nucleic acid and the tertiary amine of the ionisable lipid [6] as well as aiding in destabilising the endosomal membrane, resulting in increased payload release and improved transfection efficiencies [4]. DSPC, along with cholesterol, constitutes nearly 50% of the formulation. Cholesterol exhibits a planar, rigid structure that allows it to ‘fill the gaps’ between phospholipid molecules in the LNP, thereby enhancing structural integrity [4] and increasing biocompatibility [7]. Increased cholesterol levels have also been shown to affect LNP structure and protein expression levels [8, 9]. The inclusion of DSPC at approximately 10% and cholesterol at around 30–40% has become a common feature of LNP formulations in both pre-clinical and clinical studies [10, 11].

Ionisable lipids are included to bind the nucleic acid payload and typically comprise most of the formulation (approximately 50%). The high molar percentage of ionisable lipids has been well documented in early siRNA-LNP research [12] and in more recent mRNA-LNP research [10]. Decreasing levels of ionisable lipids reduce binding capacity for the mRNA payload, which may affect LNP efficacy, as protein expression from mRNA depends on the amount of mRNA molecules that reach the translational machinery. Finally, LNPs also contain 1.5% PEGylated lipid, which provides stability and prevents aggregation [4, 7]. PEGylated lipids can also affect LNP particle size, with higher concentrations yielding smaller particles [13]. It has also been observed that increased PEGylation can lead to increased mRNA expression, possibly due to longer circulation times and greater stability. However, the inclusion of PEGylated lipids also raises concerns about anti-PEG antibodies, which may cause allergic reactions and reduce overall transfection efficacy [14].

Despite the clinical success of current LNP formulations, there remains a need to revisit and challenge the accepted lipid molar ratio, particularly to improve extrahepatic targeting and enhance transfection efficacy. In parallel, there is a clear need for robust, scalable, and reproducible strategies for formulation screening at the bench scale. Traditionally, formulation optimisation is undertaken using one-factor-at-a-time (OFAT) approaches, in which one factor is varied while the others remain constant. OFAT approaches offer a practical and straightforward means of systematically analysing individual parameters; however, given the complexity of LNP systems and the extent of lipid-lipid interactions, this approach may overlook interaction effects and constrain exploration of the formulation design space [3]. Furthermore, the restricted experimental space inherent to OFAT can lead to repeated testing and fragmented interpretation, thereby limiting insight into formulation interdependencies.

These limitations emphasise the need for more robust and scalable formulation strategies, including Design of Experiments (DoE). DoE provides a systematic framework that maximises information gained from a reduced number of formulations [3] and enables the identification of interaction effects that are inaccessible using OFAT approaches. However, while DoE can provide broader insight into formulation behaviour, its ability to predict complex biological outcomes remains limited, as relationships identified using in vitro assays do not always translate to in vivo performance. This disconnect has been reported for nucleic acid nanoparticle systems, where DoE-optimised formulations showed poor correlation between in vitro transfection efficiency and in vivo expression, highlighting the challenges of applying statistically optimised models to multifactorial biological systems [15].

Therefore, this study directly compares OFAT and DoE approaches within the same LNP system, focusing on how conclusions regarding lipid molar ratio, physicochemical properties, and in vitro and in vivo mRNA expression depend on both the formulation strategy and the manufacturing conditions employed. In addition, manually executed and automated microfluidic approaches were evaluated to assess the influence of process conditions on formulation–performance relationships. By revisiting the established lipid ratio paradigm, this work provides comparative insights into formulation screening strategies, while contributing to the existing knowledge on how molar ratios affect LNP physicochemical properties and in vitro/in vivo protein expression.

## Materials and methods

### Materials

The phospholipid 1,2-distearoyl-*sn*-glycero-3-phosphocholine (DSPC) and the PEGylated lipid 1,2-dimyristoyl-*sn*-glycero-3-methoxypolyethylene glycol-2000 (DMG-PEG2000) were acquired from Avanti Polar Lipids (Alabaster, AL, USA), while the ionisable lipid Heptadecan-9-yl 8-((2-hydroxyethyl)[6-oxo-6-(undecyloxy)hexyl]amino) octanoate (SM-102) was purchased from Broadpharm (San Diego, CA, USA). Heat-inactivated fetal bovine serum (FBS), cholesterol, polyadenylic acid (Poly A), sodium citrate dihydrate, citric acid and HPLC-grade methanol were obtained from Merck Life Science UK Ltd. (Gillingham, UK). Ethanol was purchased from Supelco (Bellefonte, PA, USA). The nucleic acid EZ Cap™ Firefly Luciferase mRNA (5-moUTP) was purchased from APExBIO (Houston, TX, USA). The Quanti-iT Ribogreen RNA Assay kit, triethylamine acetate buffer, and Tris–HCl buffer were purchased from Thermo Fisher Scientific (Invitrogen, Waltham, MA, USA). Cell culture reagents, including Minimal Essential Medium (MEM), sodium pyruvate, penicillin/streptomycin, and TrypLE, were purchased from Gibco Technologies, while HEK-293 cells were obtained from the American Type Culture Collection (ATCC) (CRL-1573). Finally, the Rabbit Reticulocyte Lysate Kit and ONE-Glo™ Luciferase Assay System were purchased from Promega Ltd. (Chilworth, UK). Deionised and ultrapure water were supplied through an in-house system.

### Microfluidic manufacture of lipid nanoparticles (LNPs)

#### One-factor at a time (OFAT)

OFAT LNPs were manufactured using the Ignite™ (Precision Nanosystems Inc, Vancouver, Canada) using a flow rate ratio (FRR) of 3:1 (aqueous:lipid) and a total flow rate (TFR) of 15 mL/min. The aqueous phase consisted of either Firefly Luciferase mRNA (Fluc) or Poly A in 50 mM citrate buffer at pH 5. The lipid phase consisted of DSPC, cholesterol, SM-102, and DMG-PEG2k at varying molar ratios (Table 1) in ethanol. LNPs were produced at a w/w ratio of 22:1 (lipid:mRNA), which corresponded to a final concentration of 0.055 mg/mL payload and 1.25 mg/mL lipid. For in vivo studies, formulations were adjusted to account for encapsulation efficiency, and all LNPs were normalised to a final mRNA concentration of 0.1 mg/mL prior to administration.

**Table 1:** Lipid compositions of LNP formulations manufactured during OFAT Testing. Each formulation is assigned an LNP number for easy identification, and individual lipid proportions are detailed in the remaining columns

LNP Number	DSPC (%)	Cholesterol (%)	SM-102 (%)	DMG-PEG2k (%)
LNP1	10	38.5	50	1.5
LNP2	20	28.5		
LNP3	30	18.5		
LNP4	40	8.5		
LNP5	48.5	-		
LNP6	-	48.5		
LNP7	20	38.5	40	
LNP8	30		30	
LNP9	40		20	
LNP10	60		-	

#### Manually executed microfluidic mixing design of experiments (mDoE)

mDoE LNPs were manufactured using the NanoAssemblr Benchtop^©^ (Precision Nanosystems Inc, Vancouver, Canada) at a FRR of 3:1 (aqueous:lipid) and TFR of 15 mL/min. The aqueous phase contained Firefly Luciferase mRNA (Fluc) in 50 mM, pH 5 citrate buffer, while the lipid phase consisted of DSPC, cholesterol, SM-102, and DMG-PEG2k at varying molar ratios (Table 2). LNPs were produced at a constant w/w ratio of 22:1 (lipid:mRNA), which corresponded to a final concentration of 0.025 mg/mL mRNA and 0.55 mg/mL lipid.

**Table 2:** Lipid compositions for LNPs manufactured during DoE testing. LNPs can be identified using the LNP number followed by ‘m’ to indicate they were manufactured manually or ‘a’ to indicate automated manufacture

LNP Number	DSPC (%)	Cholesterol (%)	SM-102 (%)	DMG-PEG2k (%)
LNP1m/a	24.38	40.53	34.17	0.92
LNP2m/a	14.13	51.53	33.42	0.92
LNP3m/a	24.13	40.53	33.42	1.92
LNP4m/a	9.50	40.00	50.00	0.50
LNP5m/a	24.39	40.53	33.42	1.67
LNP6m/a	9.50	58.00	30.00	2.50
LNP7m/a	30.00	39.50	30.00	0.50
LNP8m/a	14.13	50.53	33.42	1.92
LNP9m/a	9.50	38.00	50.00	2.50
LNP10m/a	15.13	40.53	43.42	0.92
LNP11m/a	9.50	60.00	30.00	0.50
LNP12m/a	30.00	38.00	30.00	2.00
LNP13m/a	29.50	38.00	30.00	2.50
LNP14m/a	14.13	41.53	43.42	0.92
LNP15m/a	11.50	38.00	50.00	0.50
LNP16m/a	18.78	43.06	36.83	1.33
LNP17m/a	14.13	40.53	43.42	1.92
LNP18m/a	30.00	38.00	31.50	0.50
LNP19m/a	24.38	41.28	33.42	0.92

#### Automated design of experiments (aDoE)

aDoE LNPs were manufactured using the Sunscreen™ microfluidic mixer with a Sunny 190XT T-mixing channel (Unchained Labs, California, USA) at a FRR of 3:1 (aqueous:lipid) and a TFR of 12 mL/min. This microfluidic platform differs from the NanoAssemblr Benchtop in channel geometry, flow dynamics, and post-processing steps, which were maintained as per manufacturer protocols. The aqueous phase consisted of Firefly Luciferase mRNA in 50 mM pH 5 citrate buffer, while the lipid phase again consisted of DSPC, cholesterol, SM-102 and DMG-PEG2k at varied molar ratios (Table 2). LNPs were manufactured at a w/w ratio of 22:1 (lipid:mRNA) corresponding to 0.04 mg/mL mRNA and 0.9 mg/mL lipid, which were concentrated 4 × using spin columns to achieve 0.16 mg/mL mRNA and 3.6 mg/mL lipid.

### Buffer exchange and purification

LNPs purified using centrifugal filtration with Amicon Ultra-15 spin columns were diluted 1:40 (v/v) with 10 mM Tris buffer at pH 7.5 and then subjected to centrifugal filtration at 2000 × g at 4 °C until the desired volume was recovered. Some LNPs were further concentrated (2 × or 4x) for in vivo and aDoE studies. LNPs purified using dialysis were placed in a dialysis membrane with a 14 kDa molecular weight cut off and dialysed against 10 mM pH 7.5 Tris buffer under magnetic stirring for 4 h at room temperature (poly A-LNPs) or 4 °C (mRNA-LNPs).

### LNP physicochemical characterisation

LNPs were characterised using both the Malvern Zetasizer Ultra and the Zetasizer ZS, with a 173° backscattering angle, with dynamic light scattering (DLS) used to measure size/polydispersity and electrophoretic light scattering (ELS) to measure zeta potential. LNP size and polydispersity were measured in a ZEN0040 micro-cuvette at a lipid concentration of 0.1 mg/mL, using citrate buffer (post-microfluidic) and Tris buffer (post-purification). The viscosity and refractive index of both diluents were 1.28 cP and 1.47, and 1.0037 cP and 1.337, respectively. Zeta potential measurements were carried out using a DTS1017 folded capillary cell at a lipid concentration of 0.1 mg/mL in water with a viscosity and refractive index of 0.8872 cP and 1.33.

### Quantification of nucleic acid encapsulation

Nucleic acid encapsulation was quantified using the Quanti iT™ Ribogreen™ RNA Assay Kit (Invitrogen™). Standard curves were prepared over appropriate concentration ranges for each nucleic acid payload. Briefly, samples were diluted to a payload concentration of 3 μg/mL, mixed with either 50 μL of 2% Triton-TE or 1X TE buffer, then incubated for 15 min at 37 °C. Fluorescence was measured using the Glomax (Promega) at excitation 475 nm and emission 500–550 nm.

### In vitro luciferase assay

HEK293 cells were seeded in a white, clear-bottom 96-well plate, at a density of 1 × 10^5^ cells/well in minimum essential media (MEM) (10% Foetal Bovine Serum, 1% sodium pyruvate, 1% penicillin streptomycin) and incubated for 48 h at 37 °C, 5% CO_2_. Following incubation, LNPs were serially diluted in MEM (mRNA concentrations of 2 µg/mL, 1 µg/mL, 0.5 µg/mL, and 0.25 µg/mL), and 100 µL of each concentration was added to the wells in triplicate, which were then incubated for a further 24 h under the same conditions. After incubation, 100 µL of ONE-glo™ luciferase substrate was added to all wells; a non-treated control was included, and luminescence was measured using a GloMax (Promega).

### Cell-free protein expression

Cell-free protein expression (CFPE) was analysed using the Rabbit Reticulocyte Lysate System (Promega). mRNA was extracted from LNPs using a two-step ethanol precipitation protocol. LNP samples were diluted to 10 µg/mL mRNA in Tris buffer, combined with ethanol and sodium acetate, and centrifuged at 14,000 rpm for 20 min. Ethanol precipitation was carried out twice then the resulting pellet was washed and resuspended in RNase-free water. Extracted mRNA was diluted to 2 µg/mL and added to reaction mixtures as detailed in Table 3. All samples were normalised to equivalent mRNA input prior to analysis. Reaction mixtures were incubated at 37 °C for 1.5 h, then terminated by placing the samples on ice. Protein expression was quantified by mixing reaction mixtures 1:1 (v/v) with ONE-Glo™ luciferase substrate and measuring luminescence using a GloMax (Promega).

**Table 3:** CFPE reaction mixtures. This table details the required component quantities for the cell-free protein expression reaction used to monitor protein expression in extracted mRNA samples

Component	Standard reaction	Positive control	Negative control
Rabbit reticulocyte lysate	35 µL	35 µL	35 µL
Amino acid mixture minus leu	0.5 µL	0.5 µL	0.5 µL
Amino acid mixture minus met	0.5 µL	0.5 µL	0.5 µL
RNasin^®^ ribonuclease inhibitor	1 µL	1 µL	1 µL
Firefly luciferase mRNA	-	2 µL	-
Extracted firefly luciferase mRNA	2 µL	-	-
RNase free water	11 µL	11 µL	13 µL

### In vivo studies

For the OFAT LNPs, to evaluate the in vivo biodistribution and expression efficiency of the mRNA-LNPs, 12–17-week-old male BALB/c mice were injected bilaterally with 50 µL/leg of mRNA-LNPs intramuscularly equating to 10 µg of firefly luciferase mRNA per mouse. The mice were then subcutaneously injected with VivoGlo D-Luciferin (Promega, UK) at a concentration of 30 mg/mL (5 µL/g), and bioluminescent signals were detected using the IVIS Spectrum (PerkinElmer). At the 6 h time point after intramuscular injection, mice were once again imaged under the bioluminescence filter following subcutaneous injection with VivoGlo D-luciferin substrate, and bioluminescence signals were again measured under the bioluminescence filter.

For the aDoE LNPs, 16–17-week-old male BALB/c mice were used to evaluate in vivo biodistribution. Each mouse received the LNPs via an intramuscular injection in the leg. Mice received 50 µL/leg of LNPs, with each leg receiving a distinct LNP formulation. Mice were then injected subcutaneously with D-luciferin and imaged 10 min later. Imaging was repeated at 6 h post-injection. A control mouse that did not receive LNPs was included for baseline comparison. Bioluminescence signals were quantified using Living Image Software (v4.0). Differences between OFAT and aDoE studies reflect variations in formulation concentration and experimental design required for each dataset.

### High-performance liquid chromatography – evaporated light scattering detector (HPLC-ELSD)

Lipid quantification was performed using a previously reported HPLC-ELSD method [16]. LNP samples were analysed using the Shimadzu HPLC-ELSD III system. Samples were injected at 10 µL with a flow rate of 0.5 mL/min. Lipids were separated using a Waters XBridge^®^ C18 column (3 mm × 150 mm, 130 Å pore size, 3.5 μm particle size) with a detector temperature of 50 °C and column oven at 45 °C. Mobile phase A consisted of 0.01 M triethylamine acetate in water, and mobile phase B consisted of 0.01 M triethylamine acetate in ethanol. A binary gradient was applied, increasing mobile phase B from 70 to 100% over 0–7 min, then returning to the initial conditions. Samples were diluted 1:5 (v/v) in mobile phase B prior to analysis. Calibration curves were prepared for each lipid. This method was used to assess lipid recovery and to confirm the formulation composition of manually prepared LNPs.

### Design of experiments (DoE)

Minitab was used to construct a mixture DoE, ensuring that the total lipid composition was constrained to 100% and that the run order was randomised. The four input parameters were DSPC (9.5–30%), cholesterol (38–60%), SM-102 (30–50%), and DMG-PEG2000 (0.5–2.5%). The design generated 19 formulations, and an additional formulation representing the standard molar ratio was included as a test standard (aDoE only). Response variables included particle size, PDI, zeta potential, encapsulation and in vitro/in vivo protein expression.

### Statistical analysis

Statistical significance was assessed using Minitab (version 19.2). Parametric data were analysed using ANOVA followed by Tukey post-hoc test, while non-parametric data were analysed using a Kruskal–Wallis test followed by Dunn's post-hoc test. All results are presented as mean ± standard deviation (SD) or standard error of the mean (SEM), as specified in figure legends, with corresponding sample sizes (n) indicated. For the DoE models, predicted accuracy was defined as agreement between predicted and experimentally measured values within 80–120%. This threshold was selected as a pragmatic range to account for experimental and biological variability while enabling meaningful assessment of model performance.

## Results

### The impact of molar ratio on LNP physicochemical properties

**Fig. 2:**
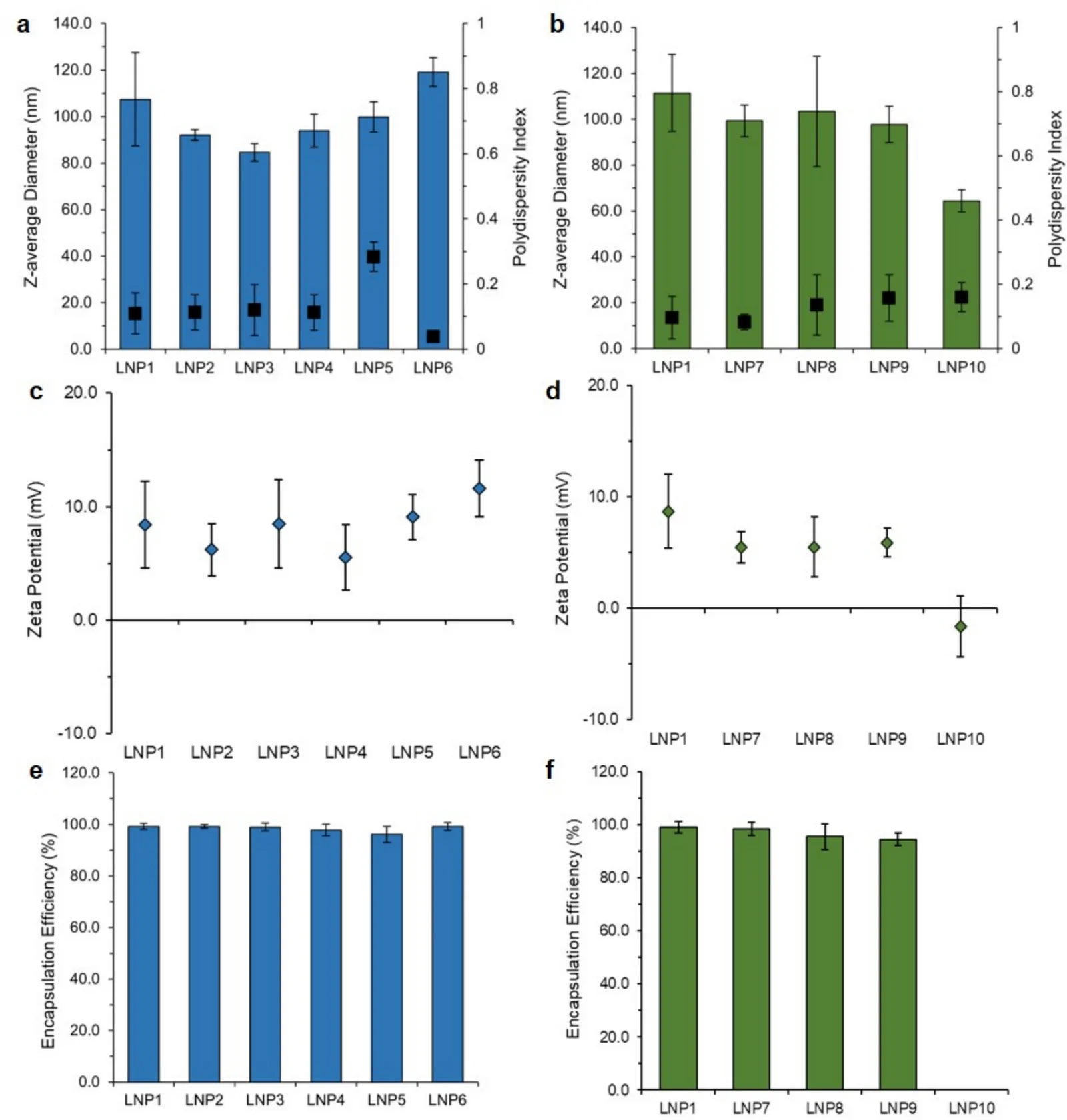
Physicochemical Characterisation and mRNA encapsulation of Group One and Group Two LNPs. LNPs were composed of DSPC:cholesterol:SM-102:DMG-PEG2000, including a standard formulation (10:38.5:50:1.5%) and variants in which DSPC content was increased from 20 to 60% at the expense of either cholesterol (Group One; LNP2–6) or SM-102 (Group Two; LNP7–10), while DMG-PEG2000 was maintained at 1.5%. Poly A was encapsulated in all formulations. (**a, b**) Particle size (bars) and polydispersity index (points) for group one (**a**) and group two (**b**). (**c, d**) Zeta potential for group one (**c**) and group two (**d**). (**e, f**) mRNA encapsulation efficiency for group one (**e**) and group two (**f**). Data are presented as mean ± standard deviation (*n* = 3–4)

Due to the limited systematic exploration of mRNA-LNP formulations with varied lipid molar ratios, the effects of altering the standard molar ratio on critical quality attributes (CQAs) and both in vitro and in vivo performance remain incompletely characterised. To address this gap, an OFAT approach was applied, varying DSPC content at the expense of either cholesterol (group one; LNP1–6) or SM-102 (group two; LNP7–10), relative to a standard formulation (LNP1; 10:38.5:50:1.5%) (Fig. 2).

Figure 2 demonstrates that, across both groups, most formulations produced Poly A-loaded LNPs in the 80–120 nm size range with low polydispersity (PDI < 0.2), indicating consistent particle formation across the tested compositional space (Fig. 2a,b). For group one (LNP1–6), increasing DSPC at the expense of cholesterol resulted in relatively minor changes in particle size and PDI. The largest LNP size was observed for LNP6, in which DSPC was removed and cholesterol content increased; however, this size increase was not significantly different from that of the standard formulation (LNP1) (Fig. 2a). In contrast, removal of cholesterol (LNP5) resulted in a significant increase in PDI (*p* < 0.05), indicating reduced uniformity of the particle population (Fig. 2a). For group two (LNP7–10), increasing DSPC at the expense of SM-102 also resulted in relatively consistent particle sizes for LNP7, LNP8 and LNP9, although greater variability in the size range is noted for LNP8. Furthermore, LNP10, which contained no ionisable lipid, exhibited a marked reduction in particle size (< 70 nm) compared to other formulations (*p* < 0.05) (Fig. 2b).

Zeta potential measurements showed that group one formulations (LNP1–6) exhibited similar positive charge with no significant differences between formulations (Fig. 2c). In group two, LNP7–9 also showed comparable zeta potential, whereas LNP10 (which contains no ionisable lipid) exhibited a shift towards negative zeta potential (Fig. 2d).

Encapsulation efficiency remained high (> 90%) across LNP1–9 (Fig. 2e,f), indicating robust nucleic acid incorporation across most formulations. In contrast, LNP10 showed negligible encapsulation, consistent with the absence of ionisable lipid required for nucleic acid complexation.

Based on these results, LNP1, 3, 5, 6, 8, and 9 were selected for further evaluation. Selection was based on a combination of acceptable CQAs (particle size < 120 nm, low PDI, and high encapsulation efficiency), alongside inclusion of formulations representing key compositional variations, including removal of individual lipid components and reduced ionisable lipid content, to enable targeted comparison of formulation–performance relationships in subsequent in vitro, ex vivo, and in vivo studies.

### The in vitro in vivo efficacy of down-selected LNPs

**Fig. 3:**
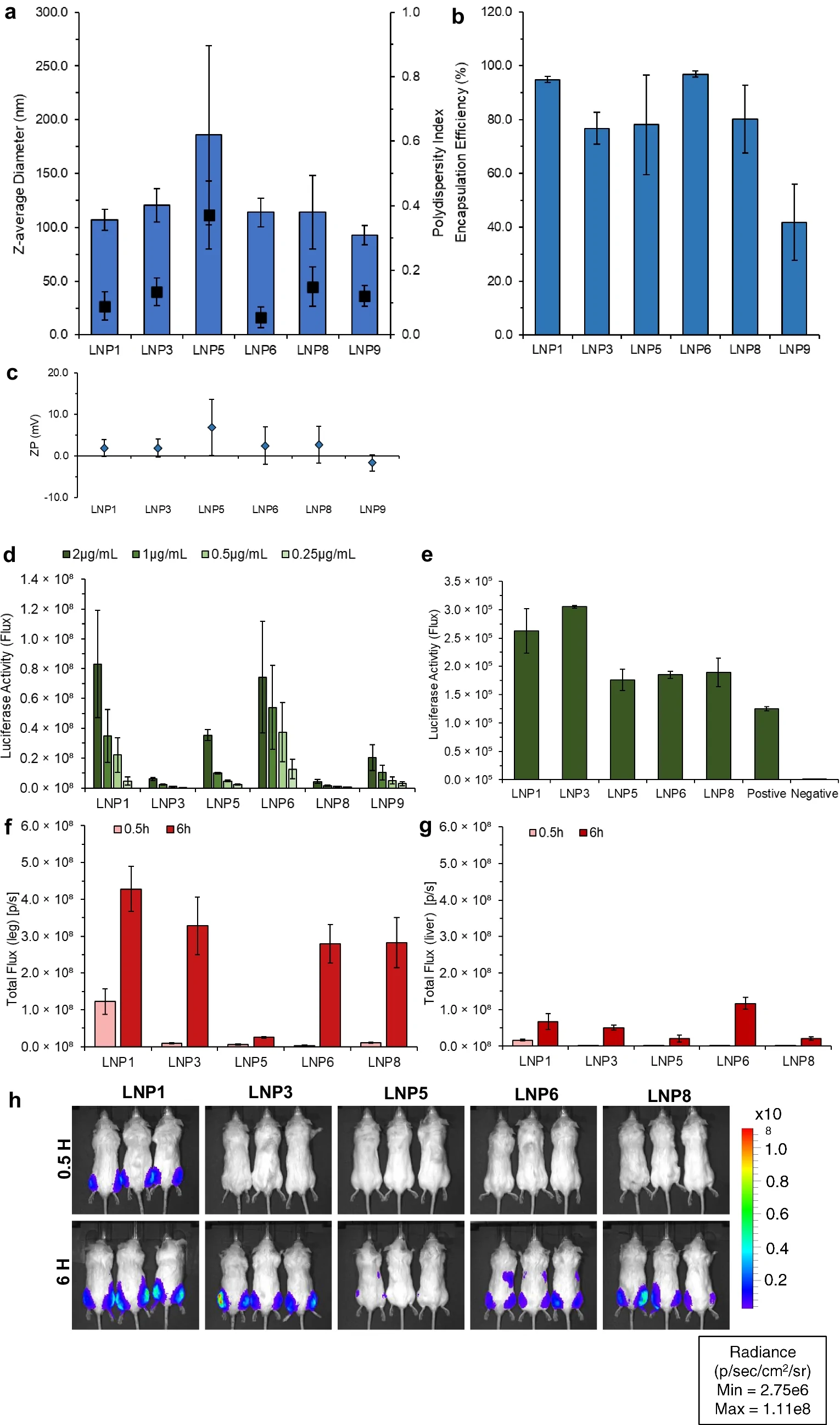
Physicochemical Characterisation and Biological Performance of Down-Selected LNP Formulations. LNPs composed of DSPC:cholesterol:SM‑102:DMG‑PEG2000 at varied molar ratios (LNP1, 3, 5, 6, 8 and 9; see Table 1) were evaluated. (**a**–**c**) Z‑average diameter, polydispersity index (PDI), mRNA encapsulation efficiency, and zeta potential. (**d**) In vitro protein expression measured as luciferase activity. (**e**) Ex vivo protein expression measured using a cell‑free protein expression (CFPE) system. (**f**, **g**) Quantified bioluminescence signals at 0.5 h and 6 h post‑intramuscular injection at the injection site (f) and liver (g). The colour scale represents luminescence intensity from 0.2 × 10⁸ (blue; minimum radiance 2.75 × 10⁶ p/s/cm^2^/sr) to 1.0 × 10⁸ (red; maximum radiance 1.11 × 10⁸ p/s/cm.^2^/sr). Representative IVIS images are shown in (**h**). Physicochemical data are presented as mean ± SD (*n* = 2–4); in vitro and ex vivo data as mean ± SEM (*n* = 2–4); in vivo data as mean ± SEM from two independent batches (*n* = 12 injection site; *n* = 6 liver)

Following down-selection, LNP formulations encapsulating Firefly luciferase mRNA (Fluc) were manufactured and evaluated for physicochemical properties, in vitro expression, cell-free protein production, and in vivo performance (Fig. 3). The selected formulations represent targeted variations in lipid composition relative to the standard molar ratio (LNP1), including reduced cholesterol (LNP3), complete cholesterol removal (LNP5), removal of DSPC (LNP6), and reduced ionisable lipid content (LNP8, 30%; LNP9, 20%) (Table 1).

Physicochemical characterisation of Fluc-loaded LNPs (Fig. 3a–c) showed that most formulations maintained comparable particle size (80–120 nm) and PDI (< 0.2) to Poly A-loaded LNPs (Fig. 2a), indicating that incorporation of mRNA did not substantially alter particle formation under standard conditions. An exception was observed for LNP5 (no cholesterol), which exhibited a notable increase in particle size and PDI variability when formulated with mRNA compared to Poly A (Fig. 3a vs Fig. 2a). Encapsulation efficiencies remained high (> 94%) across LNP1–8 (Fig. 3b), consistent with Poly A-loaded formulations (Fig. 2), where both LNP8 (30% ionisable lipid) and LNP9 (20%) showed high encapsulation. However, for mRNA, encapsulation efficiency was maintained for LNP8 but was markedly reduced for LNP9 (~ 41%), suggesting that the impact of reduced ionisable lipid content may be more pronounced when encapsulating mRNA compared to Poly A. Zeta potential measurements (Fig. 3c) showed no significant differences between formulations, with all LNPs exhibiting similar surface charge profiles, consistent with Poly A-loaded formulations (Fig. 2c–d). Overall, incorporation of mRNA revealed greater formulation-dependent differences than Poly A, suggesting that the nature of the encapsulated nucleic acid can influence the extent to which compositional variations affect LNP properties.

In vitro luciferase expression (Fig. 3d) showed that LNP1 (standard molar ratio) achieved high expression across the tested concentration range. Reducing cholesterol content (LNP3) or removing it entirely (LNP5) resulted in lower expression compared to LNP1. Removal of DSPC (LNP6) resulted in expression levels comparable to those of the standard LNP1 formulation. A reduction in ionisable lipid content led to decreased expression, as observed in LNP8 (30%) and LNP9 (20%). No significant differences in cell viability were observed across formulations, indicating that changes in lipid composition did not adversely affect cellular viability under the conditions tested (data not shown). Based on reduced encapsulation efficiency and lower in vitro expression, LNP9 was excluded from further evaluation.

Cell-free protein expression was assessed using a cell-free protein expression (CFPE) system (Fig. 3e) to evaluate the translational capacity of mRNA recovered from each LNP formulation. All samples supported protein expression, indicating that mRNA remained functional following formulation and extraction. LNP1 and LNP3 exhibited higher mean expression than the positive control (naked mRNA), while LNP5, LNP6 and LNP8 showed similar expression to the positive control. Variability in CFPE output likely reflects differences in encapsulation efficiency and mRNA recovery during extraction, rather than intrinsic differences in translational capacity.

In vivo bioluminescence imaging (Fig. 3f–h) revealed formulation-dependent differences in expression profiles. At 0.5 h post-injection, LNP1 exhibited significantly higher expression at the injection site compared to LNP3, LNP5 and LNP6 (*p* < 0.05), indicating more rapid early expression from the standard formulation. By 6 h, expression levels for LNP1, LNP3, LNP6, and LNP8 were comparable, suggesting convergence of expression over time, while LNP5 (no cholesterol) remained significantly lower than all formulations (*p* < 0.01).

Analysis of systemic (liver-associated) expression showed low overall levels across formulations. At 0.5 h, LNP1 exhibited significantly higher liver expression compared to all other formulations (*p* < 0.01). By 6 h, these differences were reduced, with LNP1, LNP3, and LNP6 exhibiting comparable expression levels, while LNP5 and LNP8 remained significantly lower than LNP6 (Fig. 3g; *p* < 0.01). Representative in vivo images (Fig. 3h) illustrate the spatial and temporal distribution of expression across formulations.

### Loss of functional activity is not caused by a loss of component integrity

**Fig. 4:**
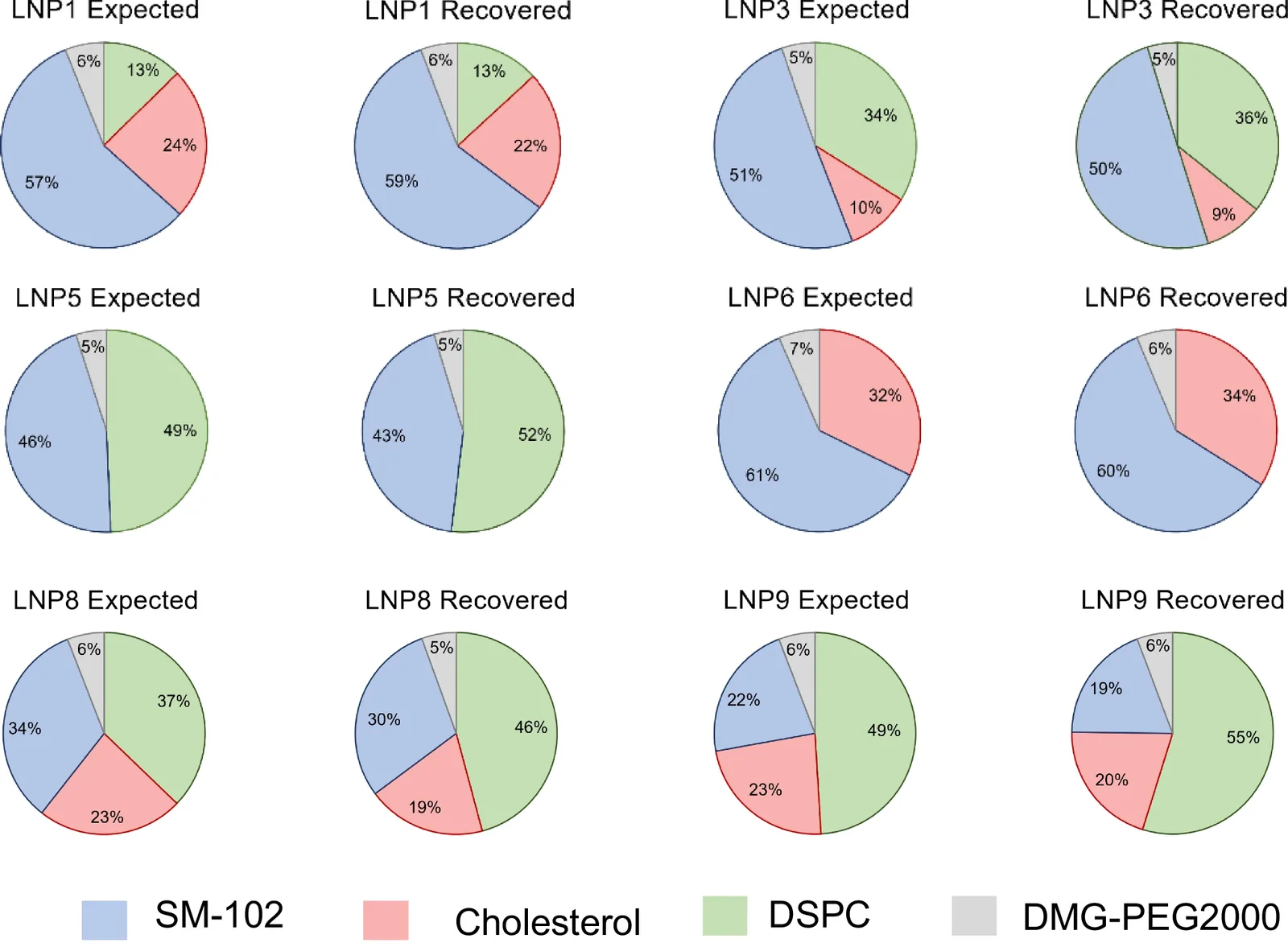
Lipid Quantification of Down-Selected LNPs. LNP formulations consist of polyA encapsulated within LNPs containing DSPC:cholesterol:SM-102:DMG-PEG2000 at varied molar ratios: LNP1 (10:38.5:50:1.5%), LNP3 (30:18.5:50:1.5%), LNP5 (48.5:0:50:1.5%), LNP6 (0:48.5:50:1.5%), LNP8 (30:38.5:30:1.5%) and LNP9 (40:38.5:20:1.5%). Results show the expected and recovered mass ratios of lipids in the LNP formulations, as determined by HPLC-ELSD. Results are presented as mean ± standard deviation (SD) (*n* = 3)

Lipid recovery and composition were quantified by HPLC-ELSD to determine whether the reduced functional activity and lack of in vitro–in vivo correlation (IVIVC) observed for selected formulations were associated with loss of component integrity. Figure 4 shows the recovered mass ratios of the LNPs, while Table 4 presents the recovery percentages for individual lipids in the formulation.

**Table 4:** Lipid recovery of individual lipids within the formulation, detected and quantified using an HPLC-ELSD protocol. Results representative of the mean ± standard deviation (SD) (*n* = 3)

	Recovered (%) ± SD				
	DSPC	Cholesterol	SM-102	DMG-PEG2k	Total
LNP1	104 ± 6	92 ± 3	103 ± 9	94 ± 5	100 ± 5
LNP3	96 ± 25	84 ± 13	89 ± 7	84 ± 21	90 ± 12
LNP5	96 ± 23	-	87 ± 17	87 ± 20	91 ± 18
LNP6	-	84 ± 7	79 ± 4	83 ± 7	81 ± 4
LNP8	130 ± 76	88 ± 13	95 ± 4	100 ± 18	106 ± 31
LNP9	95 ± 39	75 ± 14	74 ± 4	82 ± 15	85 ± 24

Total lipid recovery exceeded 80% for all formulations, ranging from 81 ± 4% (LNP6) to 106 ± 31% (LNP8), with no formulation-dependent trend (Table 4). Individual lipid recoveries were similarly consistent across formulations, with SM-102 and DMG-PEG2k recovered within ~ 74–103% and ~ 82–100%, respectively. Cholesterol recovery ranged from 75 ± 14% (LNP9) to 92 ± 3% (LNP1). Greater variability was observed for DSPC, particularly in LNP8 (130 ± 76%), although this was associated with high standard deviation.

Recovered molar ratios (Fig. 4) closely matched expected compositions for LNP1, LNP3, LNP5, and LNP6, demonstrating minimal deviation from target formulations. While increased DSPC proportions were observed in LNP8 and LNP9 relative to expected values, these shifts did not substantially disrupt the overall lipid balance.

No consistent loss of any individual lipid component was observed across formulations, and total lipid recovery remained high. As lipid recovery was confirmed to be consistent in these initial studies, and no systematic differences in biological performance were attributable to compositional loss, lipid quantification was not repeated for subsequent DoE formulations.

### Rationale for transition from OFAT to DoE

While the preceding OFAT studies provided a useful baseline for understanding how variation in lipid molar ratios influences CQAs and formulation efficacy, this approach is inherently limited in capturing interaction effects between components. Such interactions are particularly important in multicomponent systems such as LNPs. Where lipid-lipid interactions can influence physicochemical properties and biological performance. In addition, the PEGylated lipid content was fixed at 1.5% throughout the OFAT studies, restricting insight into its potential contribution to formulation behaviour and representing a notable gap in the design space explored.

To address these limitations, a DoE approach was implemented to enable a more comprehensive and statistically robust evaluation of formulation variables. A mixture DoE was selected, as this allows lipid components to be varied simultaneously while constraining the total molar composition to 100%, thereby reflecting the inherent interdependence of formulation components. This approach was specifically chosen to quantify interaction effects and to facilitate the development of predictive models for formulation optimisation.

The mixture DoE generated 19 formulations, which were manufactured using either a manually operated microfluidic mixer or an automated microfluidic mixer. The resulting formulations were denoted as mLNPs and aLNPs, respectively. Both formulation sets were characterised with respect to the key CQAs identified earlier: particle size, PDI, zeta potential, and mRNA encapsulation efficiency, as well as in vitro transfection efficacy. In addition, the aLNPs formulations were progressed to in vivo evaluation to assess their translational relevance. Experimental data were subsequently analysed using Minitab to generate predictive models and assess the significance of individual factors and their interactions. Model validation was performed to evaluate the reliability of predictions within the explored design space. Therefore, this approach enables a direct comparison with OFAT-derived insights, while providing a framework to evaluate whether increased experimental complexity translates into improved predictive power. CQA values for the manually manufactured DoE formulations (mLNPs) are summarised in Figure S1. Most formulations exhibited particle sizes below 200 nm, PDI values below 0.2, near-neutral zeta potentials, and encapsulation efficiencies greater than 75%, indicating that most formulations remained within acceptable physicochemical ranges.

### Composition–response relationships in manual DoE LNPs (mLNPs)

**Fig. 5:**
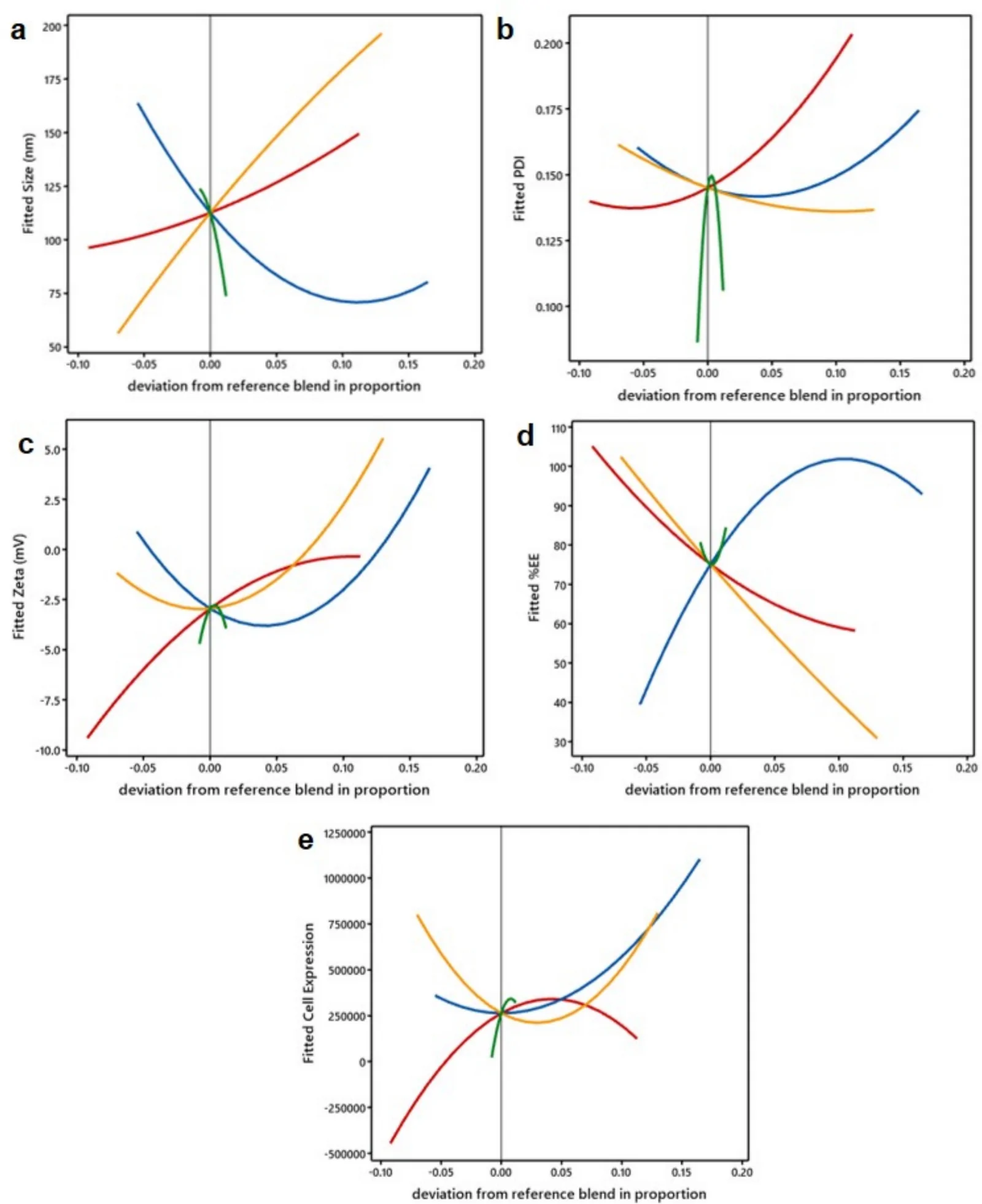
Cox Response Trace Plots for mDoE LNPs. The response trace plots demonstrate the relationship between size (**a**), PDI (**b**), zeta potential (**c**), %EE (**d**), and cell expression (Flux) (**e**) and changes in relative molar proportions of lipid components within the mixture. The reference blend is indicated as the centroid of the vertices at 18.78% DSPC, 43.06% cholesterol, 36.83% SM-102 and 1.33% DMG-PEG2K. Individual lipids are represented as coloured lines: DSPC (red), cholesterol (blue). SM-102 (yellow) and DMG-PEG2K (green). Cox Response Trace Plots were generated using Minitab

The influence of lipid composition on LNP properties was evaluated using Cox response trace plots (Fig. 5), enabling assessment of component sensitivity relative to the reference formulation. Across all responses, these plots illustrate how variation in the relative proportions of lipid components influences physicochemical properties and protein expression within the design space explored.

Particle size showed clear dependence on lipid composition (Fig. 5a). Shifts towards higher DSPC and SM-102 proportions were associated with larger particle sizes, whereas shifts towards higher cholesterol or DMG-PEG2k resulted in smaller particle sizes. In contrast to the OFAT study, where variation in DSPC and cholesterol resulted in relatively minor changes in particle size, the DoE model indicates greater sensitivity to compositional variation across the broader design space, including effects associated with DMG-PEG2k that were not explored in the OFAT approach.

PDI exhibited minimal variation across the design space (Fig. 5b). Among the lipid components, variation of DMG-PEG2k was associated with the most notable changes in PDI, while DSPC, cholesterol, and SM-102 showed more limited influence.

Zeta potential remained within a narrow near-neutral range (0 to − 7 mV) across all formulations (Fig. 5c), with only minor variation observed across the design space. Small shifts were associated with changes in DSPC proportion, while cholesterol, SM-102, and DMG-PEG2k showed minimal influence.

Encapsulation efficiency varied with lipid composition (Fig. 5d). Higher DSPC proportions were associated with reduced encapsulation efficiency, whereas higher cholesterol levels were associated with increased encapsulation efficiency. Higher SM-102 proportions were also associated with reduced encapsulation efficiency; however, as this reflects a mixture design, this trend represents the effect of increasing SM-102 relative to the other lipid components rather than its isolated contribution. DMG-PEG2k showed a limited, localised effect.

Protein expression varied non-linearly across the formulation space (Fig. 5e). Higher DSPC proportions were associated with an increase in expression near the reference composition, followed by a reduction at higher levels. Higher cholesterol proportions were associated with increased expression across the design space. SM-102 exhibited a U-shaped relationship, while DMG-PEG2k showed a localised effect around the reference composition.

### Modelling the characteristics of manual DoE LNPs (mLNPs)

Data obtained from physicochemical characterisation and expression studies were used to generate predictive models for each output using Minitab. These models were visualised as contour plots to provide a spatial representation of the composition–response relationships identified in the Cox response trace plots.

**Fig. 6:**
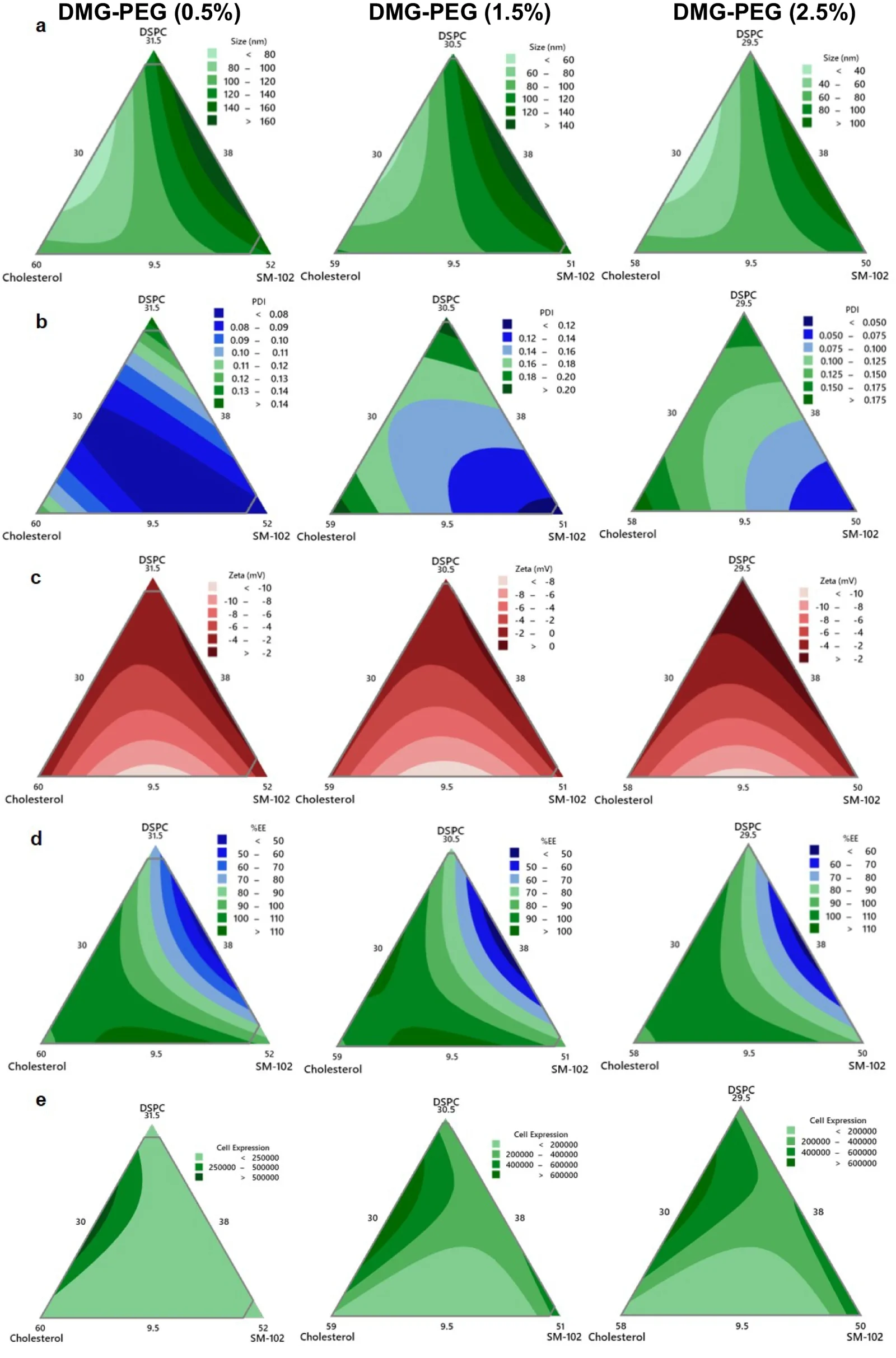
Contour Plots developed form the mLNP Model. Generated using Minitab, these contour plots demonstrate the relationship between lipid composition and (**a**) z-average diameter (nm), (**b**) polydispersity index, (**c**) zeta potential (mV), (**d**) encapsulation efficiency (%) and (**e**) in vitro protein expression in HEK-293 cells. The ternary axes represent DSPC, cholesterol, and SM-102, while DMG-PEG2k was held constant at low (0.5%), medium (1.5%) and high (2.5%) molar percentages

Figure 6 presents contour plots for each response, with DSPC, cholesterol, and SM-102 represented on the ternary axes, while DMG-PEG2k was fixed at low (0.5%), medium (1.5%) and high (2.5%) molar ratios.

Figures 6a and 6b show the predicted effects of composition on particle size and PDI. Consistent with trends observed in the Cox response trace plots, higher DMG-PEG2k proportions were associated with smaller particle sizes and lower PDI. The largest particle sizes (dark green regions) were predicted in compositions characterised by lower cholesterol and DMG-PEG2k and higher DSPC proportions. Across all DMG-PEG2k levels, PDI remained below the acceptance threshold of 0.2.

Figures 6c–e show the predicted effects of composition on zeta potential, encapsulation efficiency, and protein expression across the three DMG-PEG2k levels. The overall distribution of responses was similar across all PEG-lipid conditions, indicating that variation in DMG-PEG2k had limited impact on these outputs within the explored design space. Zeta potential (Fig. 6c) remained within a narrow near-neutral range (approximately 0 to − 10 mV), with only minor variation across compositional regions. Encapsulation efficiency (Fig. 6d) was highest in regions characterised by increased cholesterol and lower SM-102 proportions, consistent across all PEG-lipid conditions. Protein expression (Fig. 6e) showed a similar spatial distribution, with higher expression observed in regions corresponding to elevated encapsulation efficiency. These contour plots therefore provide a complementary visualisation of the composition–response relationships identified in the section "Composition–response relationships in manual DoE LNPs (mLNPs)".

### Validating mLNP DoE as a predictive model

The data were used to fit a regression model, enabling prediction of the CQAs for previously tested OFAT LNPs, thereby assessing the predictive capability of the mLNP model. Table S1 presents the predicted and actual values for OFAT LNPs, along with the prediction accuracy. Prediction accuracy was defined as values within 80–120% of the experimentally measured data.

Regression analysis was statistically significant for both mLNP size and polydispersity index (PDI) (*p* < 0.05); however, the models showed limited goodness of fit, with R^2^ values of approximately 50% (Table S3). This is reflected in the predictive modelling of the OFAT LNPs (Table S1), where the model accurately predicted LNP1, LNP6, LNP8, and LNP9, but overestimated the size of LNP3 and LNP5. Additionally, the model did not accurately predict PDI.

The regression analyses for the remaining outputs (zeta potential, encapsulation, and in vitro expression) were not statistically significant (*p* > 0.05), with R^2^ values ranging from 15 to 36% (Table S3). Accordingly, these models showed poor predictive performance for zeta potential and in vitro expression. However, the model accurately predicted encapsulation efficiency for LNP1, LNP6, and LNP8. Overall, the model demonstrated limited predictive capability and could not be robustly validated across the evaluated CQAs, prompting further investigation into whether improved process control could enhance model performance.

### Composition–response relationships in automated DoE LNPs (aLNPs)

**Fig. 7:**
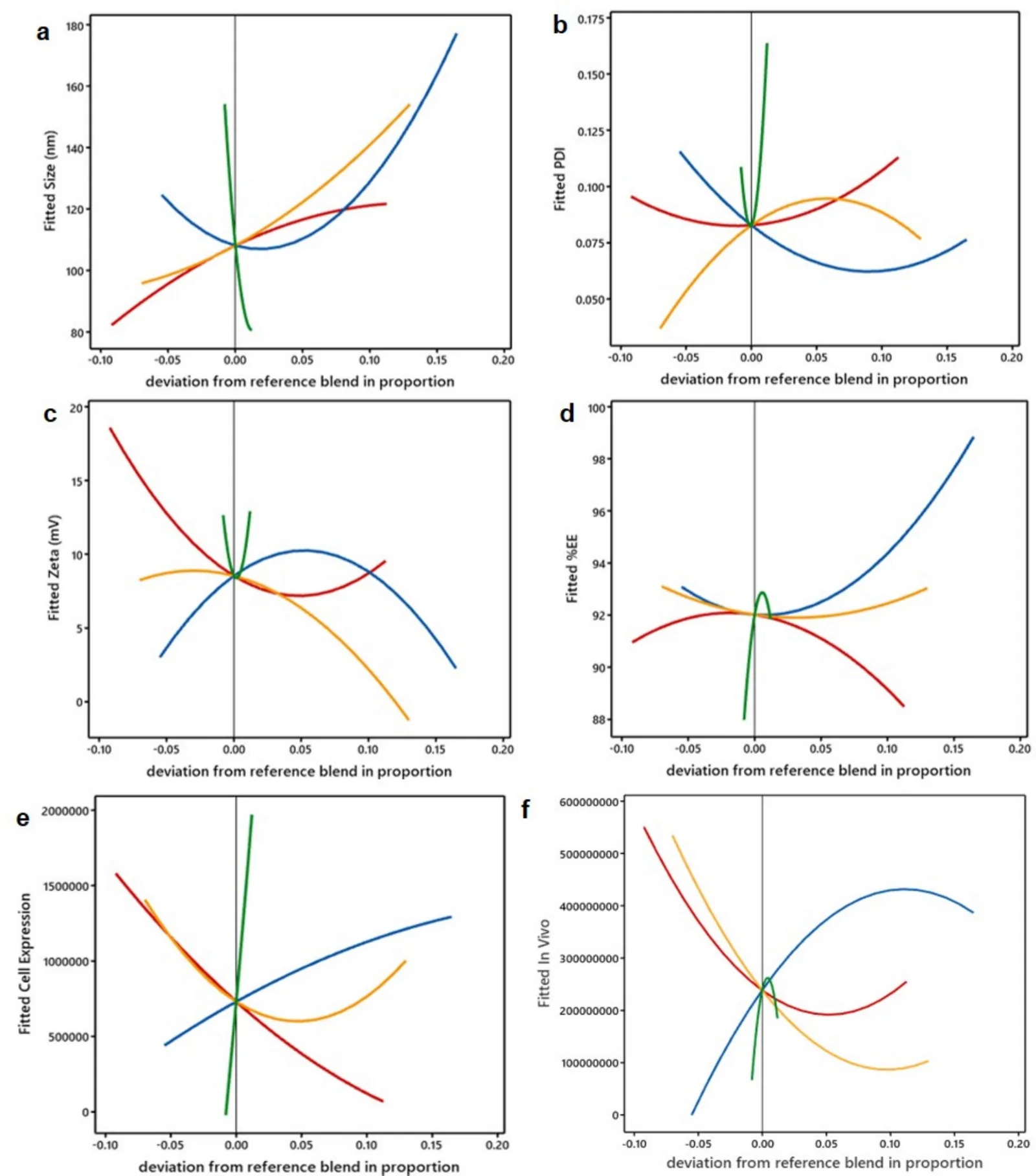
Cox Response Trace Plots for aDoE LNPs. The response trace plots demonstrate the relationship between size (**a**), PDI (**b**), zeta potential (**c**), %EE (**d**), cell expression (Flux) (**e**), and in vivo protein expression (Total Flux [p/s]) (f) and changes in the relative molar proportions of the lipid components within the mixture. The reference blend is indicated as the centroid of the vertices at 18.78% DSPC, 43.06% cholesterol, 36.83% SM-102 and 1.33% DMG-PEG2K. Individual lipids are represented as coloured lines: DSPC (red), cholesterol (blue). SM-102 (yellow) and DMG-PEG2K (green). Cox Response Trace Plots were generated using Minitab

To assess whether improved process control could enhance model performance, the DoE study was repeated using an automated microfluidic platform. The CQAs of aLNPs were analysed using the same methodology as for mLNPs, with the addition of in vivo evaluation and inclusion of the standard formulation as a control (aLNP20). Results are shown in Fig. 7, with corresponding data summarised in Figure S2.

Overall, aLNP formulations exhibited particle sizes < 206 nm, PDI < 0.2, zeta potentials between 0 and 17 mV, and encapsulation efficiencies > 79%. Particle size trends were broadly consistent with those observed for mLNPs, increasing DSPC and SM-102 proportions were associated with larger particle sizes, while increasing DMG-PEG2k resulted in smaller particles (Fig. 7a). However, in contrast to the manual model, increasing cholesterol was associated with increased particle size. The largest particles were observed for aLNP11 (~ 205 nm), which combined reduced DMG-PEG2k, increased cholesterol, and reduced SM-102 relative to the reference formulation (Figure S2). Polydispersity index remained low across all formulations, with minimal variation observed (Fig. 7b).

Zeta potential values were slightly more positive overall compared to mLNPs, ranging from 0 to 17 mV (Fig. 7c). Variation across the design space was limited, with only minor shifts observed. Increasing DSPC and cholesterol were associated with small reductions in zeta potential, while SM-102 showed a modest decreasing trend at higher proportions. DMG-PEG2k exhibited a localised effect around the reference composition. Encapsulation efficiency remained strongly dependent on lipid composition (Fig. 7d). Higher encapsulation efficiencies were observed in regions with increased cholesterol and reduced SM-102, consistent with the manual model.

Protein expression exhibited non-linear behaviour across both in vitro (Fig. 7e) and in vivo (Fig. 7f) models. In both systems, increasing cholesterol was associated with increased expression, while higher DSPC proportions were associated with reduced or marginally affected expression at higher compositions in vitro. DMG-PEG2k showed a localised effect around the reference composition. Differences were observed for SM-102: it displayed a U-shaped relationship in vitro, whereas in vivo expression increased with decreasing SM-102.

Corresponding experimental data are shown in Figure S2d–e. Two formulations, aLNP20 (standard composition) and aLNP9, demonstrated concordance between in vitro and in vivo expression. In contrast, aLNP4, which showed the highest in vivo expression, exhibited low in vitro expression and lower encapsulation efficiency.

### Modelling the characteristics of automated DoE LNPs (aLNPs)

**Fig. 8:**
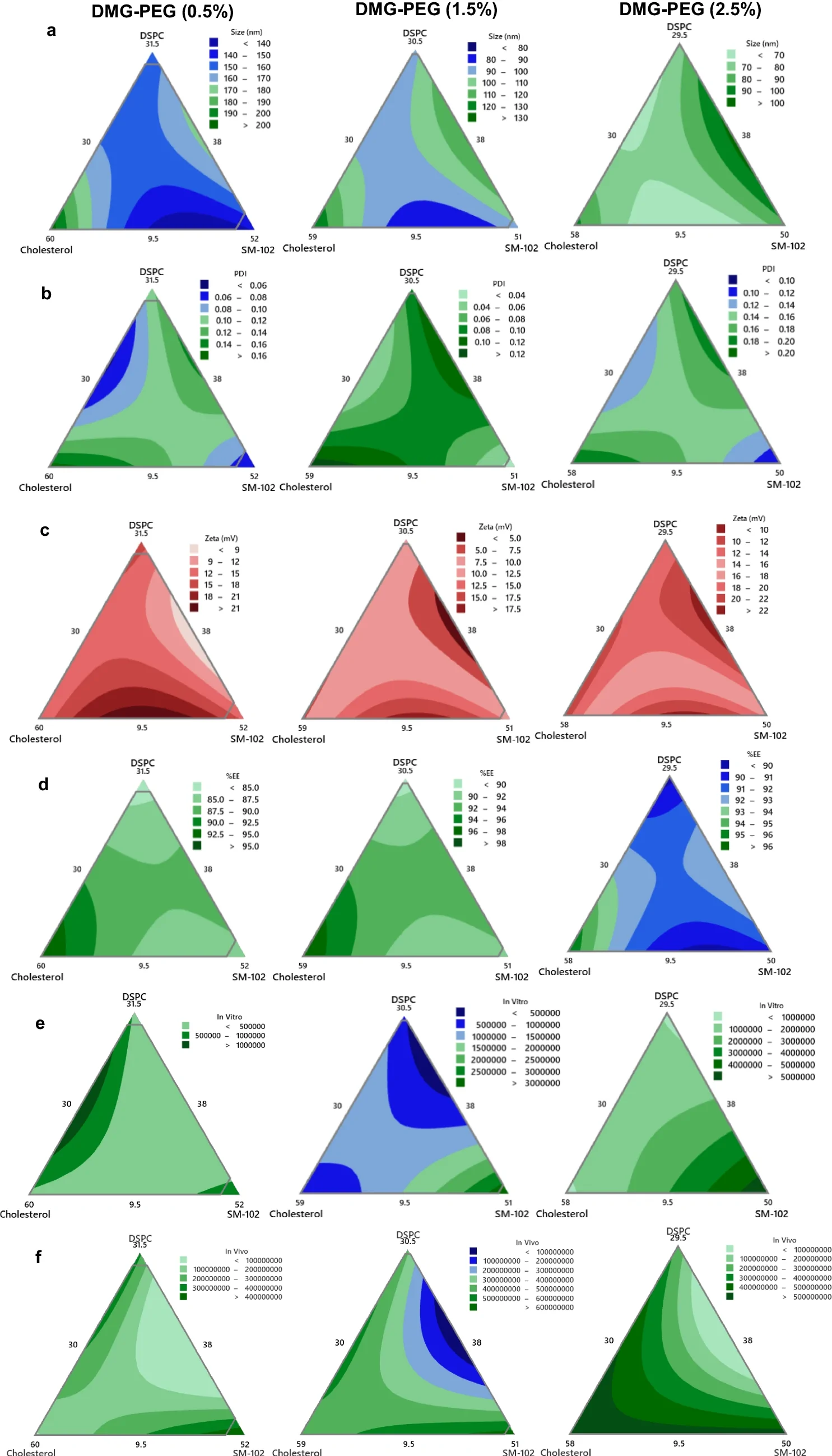
Contour Plots developed form the aLNP Model. Generated on Minitab, these contour plots demonstrate the 2D relationship between the lipids and (**a**) z-average diameter (nm), (**b**) polydispersity index, (**c**) zeta potential (mV), (**d**) encapsulation efficiency (%), (**e**) in vitro protein expression in HEK-293 cells and (**f**) in vivo protein expression in BALB/c mice. The X, Y and Z axis consist of DSPC, cholesterol and SM-102 while DMG-PEG2k was held constant at low (0.5%), medium (1.5%) and high (2.5%) molar percentages

Contour plots were generated to visualise the spatial distribution of composition–response relationships across the design space (Fig. 8), complementing the directional trends identified in the response trace plots (Fig. 7). Figures 8a and 8b highlight distinct compositional regions associated with smaller and more homogeneous particles. These regions were consistently located at higher cholesterol proportions and reduced SM-102, and were maintained across all DMG-PEG2k levels, indicating limited dependence on PEG-lipid content. Zeta potential (Fig. 8c) remained positive across the design space, with only moderate variation and no clearly defined compositional regions associated with substantially different surface charge. Encapsulation efficiency (Fig. 8d) formed a well-defined high-encapsulation region at increased cholesterol and reduced SM-102, which was preserved across all DMG-PEG2k levels. In vitro protein expression (Fig. 8e) exhibited greater spatial variability than other outputs, with multiple regions of increased expression observed across the design space. The location of these regions varied with DMG-PEG2k level, indicating sensitivity to PEG-lipid content. In contrast, in vivo protein expression (Fig. 8f) showed a more consistent spatial distribution, with higher expression localised to a single compositional region characterised by reduced SM-102 and increased cholesterol. This region was maintained across all DMG-PEG2k levels.

### Validating aLNP DoE as a predictive model

Finally, the aLNP DoE formulations were used to fit a regression model, which could be used to analyse the previous OFAT samples and to generate prediction accuracy for a range of CQAs, as shown in Table S2. The regression model analysed from the aLNP DoE was statistically significant (*p* < 0.05) (Table S3) for four outputs: LNP size, PDI, %EE, and in vitro protein expression. Particle size was predicted with the highest accuracy (R^2^ = 81.74%) (Table S3), and predictions were accurate for LNP1, LNP6, LNP8, and LNP9. PDI and encapsulation efficiency showed moderate predictive performance (R^2^ = 48% and 44%, respectively) (Table S3), with encapsulation efficiency accurately predicted for all formulations except LNP9. Finally, while in vitro protein expression has low accuracy for all LNPs, the model was able to predict which formulations would be the best-performing LNPs. In contrast, models for zeta potential and in vivo protein expression were not statistically significant (R^2^ = 5% and 43%, respectively) (Table S3). However, the in vivo model could accurately predict in vivo expression for LNP1, LNP3 and LNP8 (Table S2). Overall, predictive performance improved compared to the manual DoE model; however, predictive power remained limited for both physicochemical CQAs (particularly for particle size and encapsulation efficiency) and biological outputs, and the aLNP DoE model could not be robustly validated across all CQAs.

## Discussion

### Revisiting the lipid ratio: insights from OFAT and DoE

Despite the clinical success of nucleic acid–LNP vaccines, continued optimisation of formulation design remains essential as applications expand beyond hepatic delivery and into increasingly complex biological contexts. Current LNP formulations are typically based on established lipid ratios derived from clinically successful systems; however, these ratios are often adopted as fixed formulations rather than systematically optimised across broader compositional spaces [17, 18]. While OFAT approaches enable interrogation of individual variables [19, 20], they do not resolve interaction effects within multicomponent formulations.

This study revisited the commonly used lipid ratio in LNP formulations by directly comparing OFAT and DoE approaches to determine whether the standard formulation represents an optimal or merely acceptable composition.

The OFAT analysis indicated that the standard formulation is relatively robust, with most formulations maintaining acceptable CQAs within the explored compositional space (Figs. 2 and 3). However, DoE revealed that this ratio represents only one point within a broader compositional landscape, with multiple regions producing comparable or improved physicochemical properties and expression (Figs. 5, 6, 7 and 8). These findings indicate that LNP performance is governed by compositional balance rather than a single fixed lipid ratio.

### OFAT defines formulation boundaries but not optimal ratios

For the OFAT formulations, molar ratios were derived from the standard LNP composition, with only the structural and ionisable lipids systematically varied. The OFAT study defined the boundaries of acceptable formulation space but provided limited insight into optimal lipid ratios.

Across the OFAT dataset, most formulations maintained CQAs within acceptable ranges (Fig. 2), indicating that the standard formulation is relatively robust to compositional variation. However, increasing DSPC and removing cholesterol led to greater heterogeneity and reduced expression (Figs. 2 and 3). These observations are consistent with the established role of cholesterol in stabilising lipid packing and supporting LNP self-assembly [21].

Variation in DSPC and SM-102 produced comparatively modest and largely directional effects across CQAs within the explored compositional space. Previous studies have demonstrated that ionisable lipid content influences lipid organisation and membrane behaviour [22]; however, within the constrained OFAT space explored here, these effects were not sufficient to define optimal formulation compositions.

DSPC and cholesterol showed opposing trends in expression (Fig. 3), with reduced cholesterol associated with lower efficacy. These findings are consistent with reports that phospholipid–cholesterol interactions contribute to membrane organisation and LNP functionality [23, 24]. Overall, these data demonstrate that OFAT is effective for identifying formulation limits and directional trends but does not resolve interaction effects or define optimal lipid ratios within the broader compositional space.

### DoE reveals composition-response relationships beyond the standard ratio

In contrast to OFAT, DoE analysis revealed non-linear and interaction-driven behaviour across the formulation space, enabling identification of compositional regions associated with favourable CQAs and expression profiles (Figs. 5, 6, 7 and 8).

Across both manual and automated DoE models, cholesterol and SM-102 emerged as dominant and opposing drivers of formulation performance. Increasing cholesterol was consistently associated with improved encapsulation efficiency and protein expression, whereas increasing SM-102 had a more variable impact on these outputs within the explored mixture space. This highlights that formulation performance is governed by relative lipid balance rather than the contribution of any single component.

DMG-PEG2k primarily influenced particle size and PDI, with higher proportions associated with reduced particle diameter, consistent with previous reports describing steric stabilisation effects of PEGylated lipids [25]. However, its influence on biological performance was more localised and dependent on the surrounding lipid composition. DoE also revealed component-dependent trade-offs between physicochemical attributes and biological performance that were not apparent from OFAT analysis alone.

Comparison of manual and automated DoE models highlighted both consistent and divergent trends. While the influence of SM-102 and DMG-PEG2k was broadly consistent across models, the direction and magnitude of the effects of DSPC and cholesterol differed between models (Figs. 5, 6, 7 and 8), indicating sensitivity to processing conditions.

These differences were observed despite comparable CQA ranges between models, suggesting that formulation–process interactions contribute to the observed variability. Given that mixing conditions are known to influence LNP formation and properties [26], these findings indicate that manufacturing execution can alter apparent composition–response relationships.

Despite improved consistency in the automated system, predictive modelling remained limited. While automation improved model fit for particle size and encapsulation efficiency, prediction of biological outputs, particularly in vitro expression, remained poor (Tables S1 and S2). This demonstrates that although DoE effectively maps the formulation space and identifies optimal compositional regions, it does not necessarily yield robust predictive models for complex biological outcomes.

### Cross-method comparison of lipid-response relationships

**Fig. 9:**
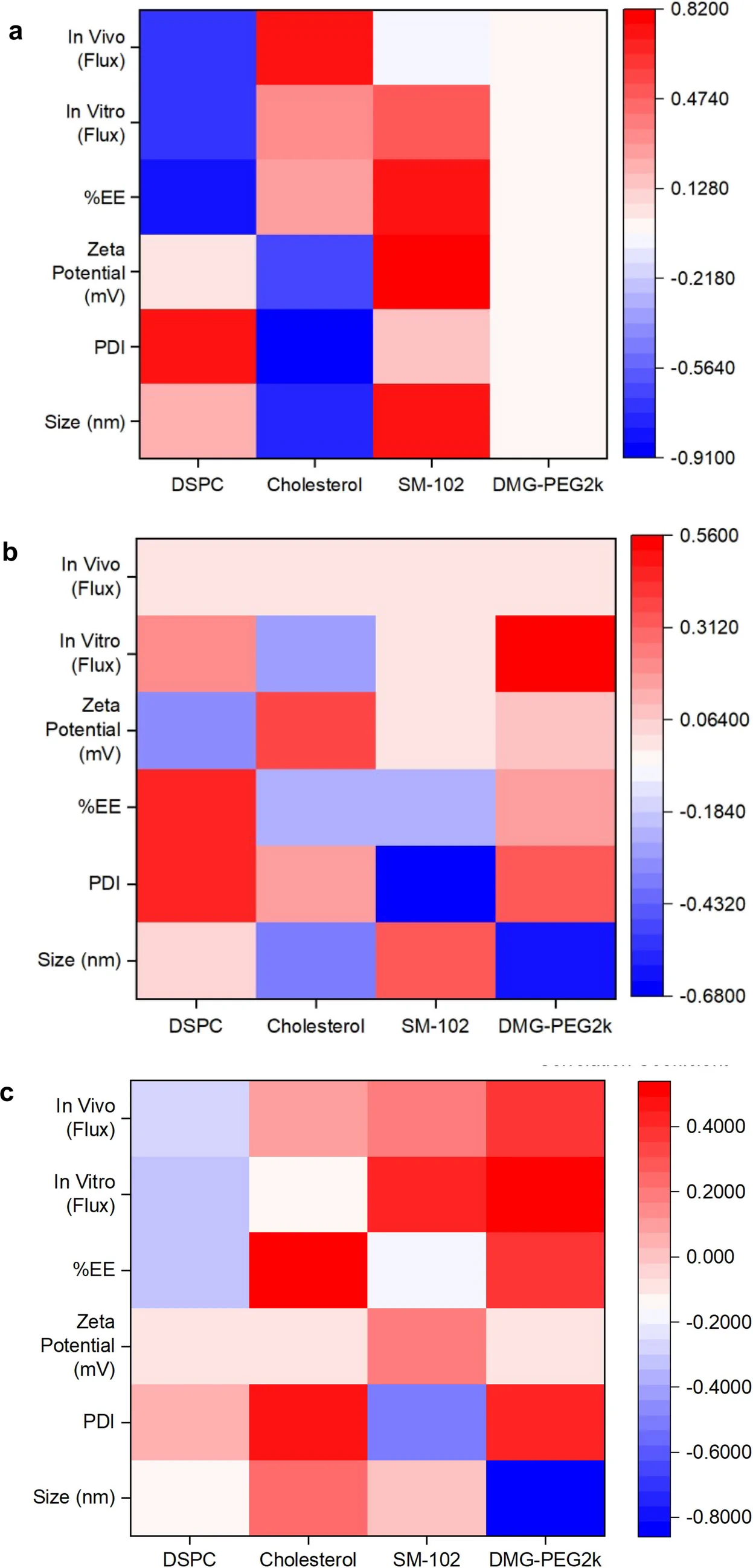
Lipid–response correlation heat maps comparing formulation strategies. Heat maps show correlation coefficients between lipid molar ratio components and measured physicochemical properties and in vitro and in vivo expression for (**a**) OFAT, (**b**) manually executed DoE (mDoE), and (**c**) automated DoE (aDoE) datasets. Colour intensity represents the strength and direction of correlation (red, positive; blue, negative). Heat maps are intended to enable comparative visualisation of lipid–response relationships across formulation strategies rather than direct quantitative prediction. Models were generated using Origin Pro 2022

To further compare lipid–response relationships across formulation strategies, correlation heat maps were used to compare lipid–response relationships across the OFAT, mDoE, and aDoE datasets (Fig. 9).

The OFAT heat map exhibited high-magnitude correlations between individual lipids and measured outputs; however, these relationships were confined to the limited compositional space explored and primarily reflected local, directional effects (Fig. 9a).

In contrast, both DoE-based heat maps showed more distributed and lower-magnitude correlations across lipid components, consistent with interaction-driven behaviour within a multicomponent formulation space. Notably, the DoE datasets exhibited broader contributions across multiple lipid components, rather than the dominant single variable observed in OFAT, consistent with previous reports highlighting the multivariate nature of LNP formulation behaviour [27] Subtle differences between the mDoE (Fig. 9b) and aDoE (Fig. 9c) heat maps suggest that manufacturing execution influences the apparent strength and direction of lipid–response relationships. Taken together, these data indicate that OFAT yields strong apparent lipid–CQA correlations within a restricted compositional space, whereas DoE reveals more distributed relationships consistent with interaction-driven behaviour.

### Implications for moving beyond a fixed lipid ratio

Previous studies have demonstrated the value of DoE approaches for interrogating lipid composition and process effects in LNP systems [27–29]. However, these approaches are often applied without direct comparison to traditional one‑factor‑at‑a‑time (OFAT) strategies. The present study addresses this gap by directly comparing OFAT and DoE within a consistent formulation framework, demonstrating that while OFAT is effective for defining formulation boundaries and confirming the robustness of the standard lipid ratio, DoE provides broader insight into non‑linear, interaction‑driven behaviour across the formulation space.

Importantly, despite these advantages, DoE did not yield robust predictive models for biological outcomes, even when implemented using an automated platform. This highlights a key limitation of composition‑based modelling approaches, indicating that formulation optimisation cannot rely on lipid ratio alone, but must also consider process conditions and biological context. Together, these findings support a shift from fixed‑ratio formulations towards region‑based optimisation strategies, in which multiple compositions within a defined design space may achieve comparable performance and emphasise the need to integrate formulation design with process control and biological evaluation.

Overall, the present work demonstrates that OFAT and DoE provide fundamentally different but complementary insights into LNP formulation behaviour, with OFAT suited to identifying directional effects and formulation boundaries, and DoE enabling systematic exploration of interaction effects and non‑linear trade‑offs. For the field, this highlights the need to move beyond inherited formulation conventions and adopt integrated optimisation strategies, with further gains expected through alignment with Quality‑by‑Design frameworks and advanced data‑driven modelling approaches [30].

## Conclusion

This study demonstrates that the conventional lipid ratio represents a robust starting point but not an optimal formulation within a broader, flexible design space. Multiple compositions achieved comparable physicochemical properties and biological performance, highlighting that LNP behaviour is governed by compositional balance rather than a single fixed ratio. While DoE enabled exploration of interaction effects and formulation trade-offs beyond OFAT, predictive performance remained limited, particularly for biological outputs, and was influenced by both formulation and processing conditions. These findings highlight a disconnect between formulation mapping and predictive capability. Moving forward, LNP optimisation should shift from fixed ratios to design-space-driven strategies that integrate composition, process, and biological context.

## Supplementary Information

Below is the link to the electronic supplementary material.

ESM 1 (PDF 666 KB)

## Data Availability

The data underpinning this publication is openly available from the University of Strathclyde Knowledge Base at [https://doi.org/https://doi.org/10.15129/dbb14e9e-561f-48a2-a326-13ab5d8c3f93].
