# Supplementary material for: Revisiting the lipid ratio in lipid nanoparticles: A comparison of OFAT and design of experiments approaches
Source: Drug Deliv Transl Res. 2026 May 18;16(10):3972–92. doi: 10.1007/s13346-026-02125-6 (PMC13619776; doi:10.1007/s13346-026-02125-6)
Supplement: Supplementary file 1 [file 13346_2026_2125_MOESM1_ESM.pdf]

Supplementary Material

Revisiting the Lipid Ratio in Lipid Nanoparticles:
A Comparison of OFAT and Design of Experiments Approaches

Jade Forrester[1], Eleanor Vaughan[2], Benjamin R. Knappett[2] and Yvonne Perrie[1]

[1]Strathclyde Institute of Pharmacy and Biomedical Sciences, 161 Cathedral St, University of Strathclyde, Glasgow, G4 0RE, UK.

[2]Unchained Labs Royston Ltd, Unit 1 Anglian Business Park, Royston, SG8 5TW, UK.

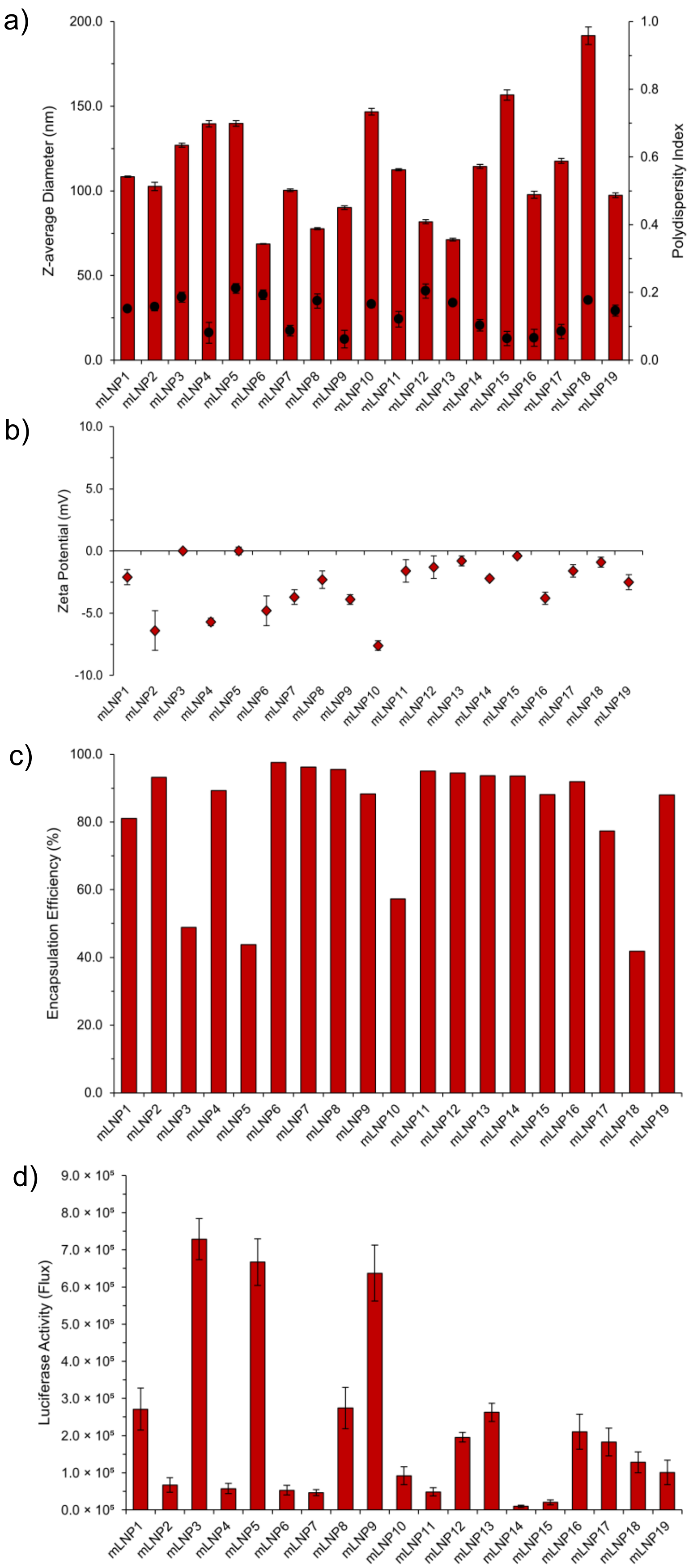

Figure S1. CQAs for mDoE LNP Formulations. (a) represents the z-average diameter (nm) (bars) and polydispersity index (circles), while (b) and (c) represent the zeta potential (mV) and encapsulation efficiency (%) respectively. (d) shows the protein expression (Flux) at a treatment concentration of 2 μg/mL mRNA for mDoE formulations. Data (excluding encapsulation efficiency) are presented as mean ± SD of three technical replicate measurements (n = 1).

Table S1. Predicted and Actual CQAs from OFAT LNPs and Predictive mLNP Model.

	Z-average Diameter (nm)			Polydispersity Index			Zeta Potential (mV)			Encapsulation Efficiency (%)			Cell Expression (Flux)		
	Predicted	Actual	Accuracy	Predicted	Actual	Accuracy	Predicted	Actual	Accuracy	Predicted	Actual	Accuracy	Predicted	Actual	Accuracy
**LNP1**	123.2	110.0	112.0	0.081	0.105	77.1	-3.6	1.8	-200.0	83.2	94.4	88.1	242848	83087484.2	0.3
**LNP3**	149.0	111.6	133.5	0.092	0.121	76.0	-1.0	1.0	-100.0	59.4	74.6	79.6	448939	5957067.5	7.5
**LNP5**	172.9	99.8	173.2	0.102	0.318	32.1	1.4	3.2	43.8	37.4	69.3	54.0	639573	35406150.8	1.8
**LNP6**	110.2	111.2	99.1	0.075	0.057	131.6	-4.9	0.7	-700.0	95.1	97.3	97.7	139803	74111959.2	0.2
**LNP8**	109.9	91.2	120.5	0.174	0.113	154.0	-1.3	0.4	-325.0	73.5	77.2	95.2	298430	4331792.5	6.9
**LNP9**	103.2	92.7	111.3	0.221	0.121	182.6	-0.1	-1.7	68.6	68.6	41.8	164.1	326221	20285874.4	1.6

Table S2. Predicted and Actual CQAs from OFAT LNPs and Predictive aLNP Model

	Z-average Diameter (nm)			Polydispersity Index			Zeta Potential (mV)			Encapsulation Efficiency (%)			Cell Expression (Flux)			In Vivo Expression (Flux)		
	Predicted	Actual	Accuracy	Predicted	Actual	Accuracy	Predicted	Actual	Accuracy	Predicted	Actual	Accuracy	Predicted	Actual	Accuracy	Predicted	Actual	Accuracy
**LNP1**	105.1	110	95.5	0.081	0.105	77.1	13.1	1.8	727.8	89.9	94.4	95.2	2411289	83087484.2	2.9	393766639	427752520	92.1
**LNP3**	76.8	111.6	68.8	0.043	0.121	35.5	13.4	1	1340.0	82.5	74.6	110.6	2251148	5957067.5	37.8	312387166	328152520	95.2
**LNP5**	50.5	99.8	50.6	0.008	0.318	2.5	13.7	3.2	428.1	75.7	69.3	109.2	2103019	35406150.8	5.9	237111153	25254186.67	938.9
**LNP6**	119.3	111.2	107.3	0.099	0.057	173.7	13	0.7	1857.1	93.5	97.3	96.1	2491359	74111959.2	3.4	434456376	278877520	155.8
**LNP8**	106.4	91.2	116.7	0.115	0.113	101.8	11	0.4	2750.0	88.7	77.2	114.9	478310	4331792.5	11.0	252570419	281874186.7	89.6
**LNP9**	107.1	92.7	115.5	0.132	0.121	109.1	9.9	-1.7	-582.4	88.1	41.8	210.8	-488180	20285874.4	-2.4			

Table S3. Model Fit Analysing of mDoE and aDoE Regression Model used for Predictions in Table S1 and S2.

	mDoE Model Fit		aDoE Model Fit	
	R^2	p-value	R^2	p-value
**Size**	51.62	0.011	81.74	<0.001
**Polydispersity Index**	51.27	0.011	47.61	0.019
**Zeta Potential**	21.72	0.284	4.66	0.864
**Encapsulation Efficiency**	16.27	0.432	44.27	0.029
**In Vitro**	35.69	0.078	54.25	0.007
**In Vivo**	N/A	N/A	18.05	0.379

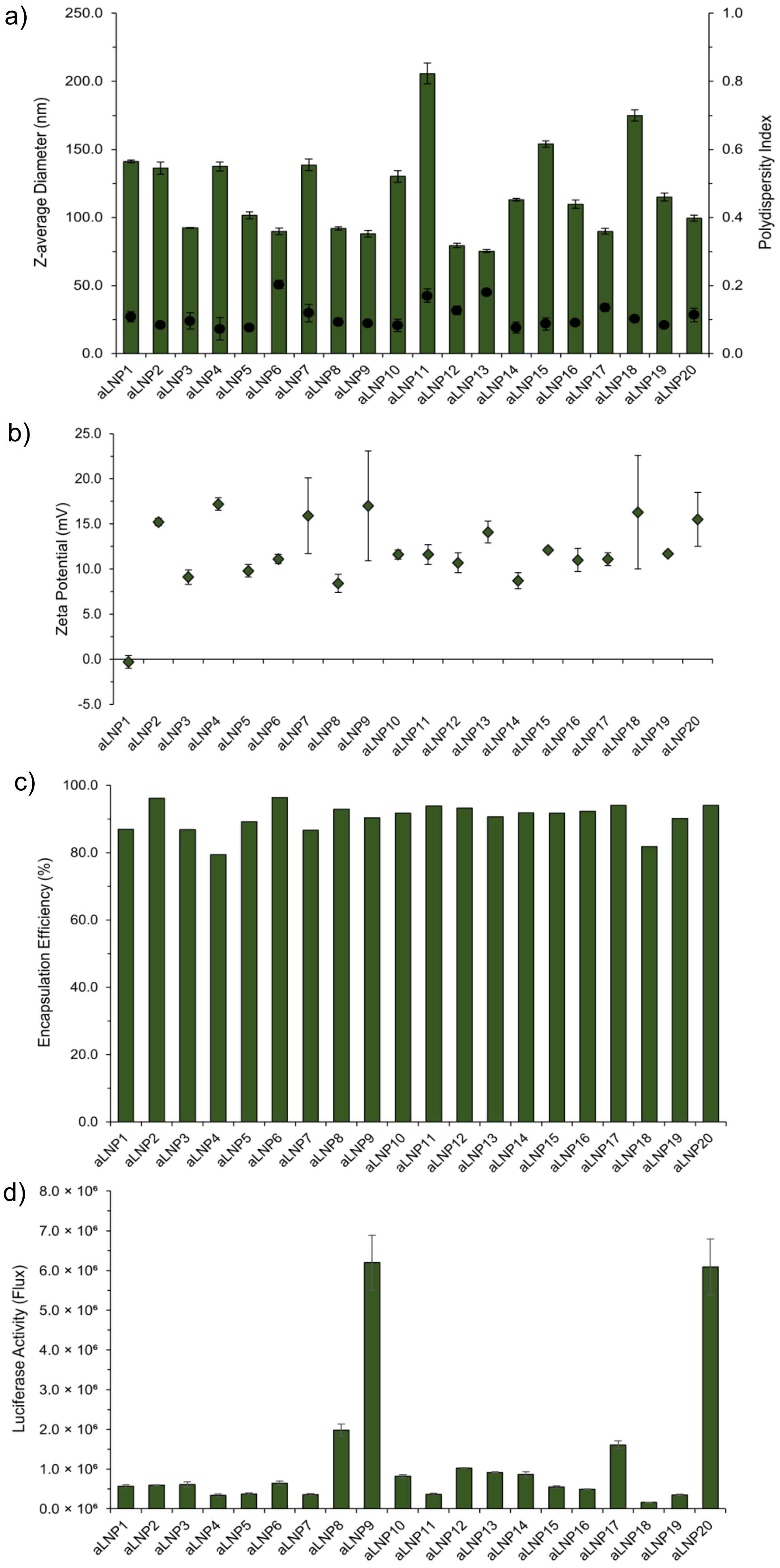

Figure S2 (continued).

e)

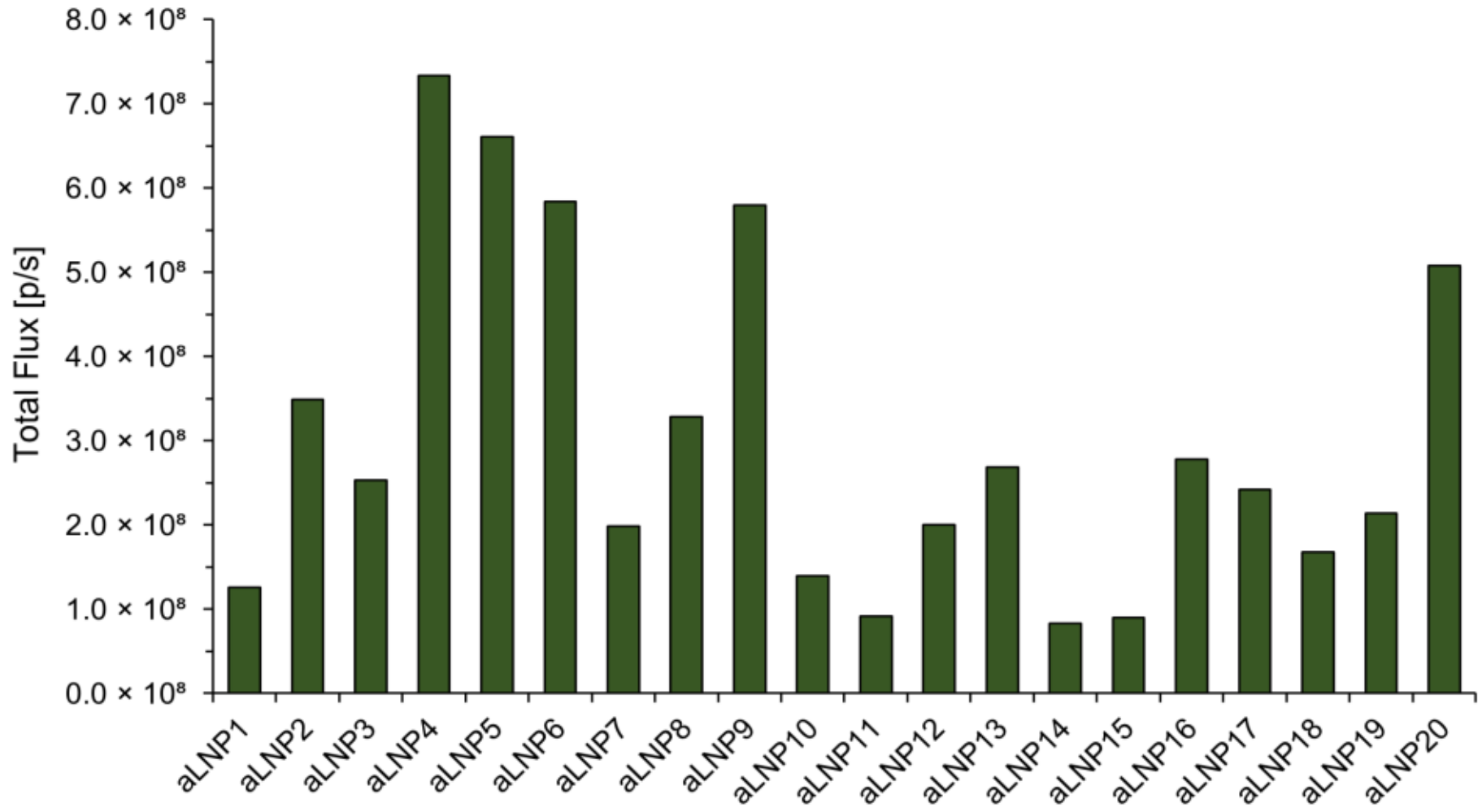

Figure S2 (continued). CQAs for aDoE LNP Formulations. (a) represents the z-average diameter (nm) (bars) and polydispersity index (circles), while (b) and (c) represent the zeta potential (mV) and encapsulation efficiency (%) respectively. (d) shows the protein expression (Flux) in HEK-293 cells at a treatment concentration of 2 μg/mL mRNA for aDoE formulations while (e) represents the protein expression (total flux [p/s]) at the injection site of BALB/c mice 6 hours post intramuscular injection with aDoE formulations. aLNP20 represents the standard molar ratio. Data (excluding encapsulation efficiency and in vivo expression) are presented as mean ± SD of three technical replicate measurements (n = 1).
